# The contributions of focused attention and open monitoring in mindfulness-based cognitive therapy for affective disturbances: A 3-armed randomized dismantling trial

**DOI:** 10.1371/journal.pone.0244838

**Published:** 2021-01-12

**Authors:** Brendan Cullen, Kristina Eichel, Jared R. Lindahl, Hadley Rahrig, Nisha Kini, Julie Flahive, Willoughby B. Britton

**Affiliations:** 1 Department of Psychiatry and Human Behavior, Brown University Medical School, Providence, RI, United States of America; 2 Department of Religious Studies, Brown University, Providence, RI, United States of America; 3 Quantitative Health Sciences Department, University of Massachusetts Medical School, Worcester, MA, United States of America; The Chinese University of Hong Kong, HONG KONG

## Abstract

**Objective:**

Mindfulness-based cognitive therapy (MBCT) includes a combination of focused attention (FA) and open monitoring (OM) meditation practices. The aim of this study was to assess both short- and long-term between- and within-group differences in affective disturbance among FA, OM and their combination (MBCT) in the context of a randomized controlled trial.

**Method:**

One hundred and four participants with mild to severe depression and anxiety were randomized into one of three 8-week interventions: MBCT (n = 32), FA (n = 36) and OM (n = 36). Outcome measures included the Inventory of Depressive Symptomatology (IDS), and the Depression Anxiety Stress Scales (DASS). Mixed effects regression models were used to assess differential treatment effects during treatment, post-treatment (8 weeks) and long-term (20 weeks). The Reliable Change Index (RCI) was used to translate statistical findings into clinically meaningful improvements or deteriorations.

**Results:**

All treatments demonstrated medium to large improvements (*ds* = 0.42–1.65) for almost all outcomes. While all treatments were largely comparable in their effects at post-treatment (week 8), the treatments showed meaningful differences in rapidity of response and pattern of deteriorations. FA showed the fastest rate of improvement and the fewest deteriorations on stress, anxiety and depression during treatment, but a loss of treatment-related gains and lasting deteriorations in depression at week 20. OM showed the slowest rate of improvement and lost treatment-related gains for anxiety, resulting in higher anxiety in OM at week 20 than MBCT (*d* = 0.40) and FA (*d* = 0.36), though these differences did not reach statistical significance after correcting for multiple comparisons (*p’s* = .06). MBCT and OM showed deteriorations in stress, anxiety and depression at multiple timepoints during treatment, with lasting deteriorations in stress and depression. MBCT showed the most favorable pattern for long-term treatment of depression.

**Conclusions:**

FA, OM and MBCT show different patterns of response for different dimensions of affective disturbance.

**Trial registration:**

This trial is registered at (v NCT01831362); www.clinicaltrials.gov.

## Introduction

Mindfulness-based programs (MBPs) are a popular approach to addressing mild to severe emotional stress, depression and anxiety [1]. However, despite the widespread application of mindfulness meditation, numerous methodological limitations preclude definitive claims about clinical effectiveness or mechanism of action [2–4]. At present, one of the largest barriers to mindfulness research is the absence of studies that dismantle multidimensional treatment packages into their most basic components and practices [2,4].

A government report concluded that central obstacle in meditation research was the lack of delineation of the different types of meditation practices, both in terms of their operational descriptions and separate (or additive) outcomes [2]. The report concluded that “further research needs to be directed toward distinguishing the effects and characteristics of the many different techniques falling under the rubric ‘meditation’” (p. 209) and called for “systematically comparing the effects of different meditation practices that research shows have promise” (p. 208).

The two most common MBPs are mindfulness-based stress reduction (MBSR) and mindfulness-based cognitive therapy (MBCT), which include under the broader umbrella term of “mindfulness” multiple meditation techniques that could be differentiated from one another in order to determine their isolated and aggregated effects [5]. These foundational MBPs draw from Buddhist formulations of meditation practice, which typically begin with concentration or tranquility practices and are followed by “insight” practices. In modern scientific research, these two practice approaches have been operationalized as focused attention (FA) and open monitoring (OM), respectively [6,7]. FA and OM are the foundation of MBPs such as MBSR and MBCT, with both programs dedicating approximately half of treatment time to FA and the other half to OM [5,6,8].

FA practice entails “voluntary focusing [of] attention on a chosen object in a sustained fashion” [7]. In addition to “directing and sustaining attention on a selected object,” one also must engage in “detecting mind wandering and distractors” and learn to disengage attention from those distractors and shift attention back to the selected object [7]. Similar to attention bias modification, FA is thought to reduce negative emotions by directing attention *away* from them to a neutral object, thereby reducing negative attentional biases that drive many affective disorders [9,10].

By contrast, OM has “no explicit focus on objects” and is further characterized by a “nonreactive meta-cognitive monitoring” of all mental contents and body sensations, regardless of valence [7]. Similar to exposure techniques, OM entails an “approach orientation” of directing attention *toward* difficult thoughts and emotions to promote affective adaptation and extinguish secondary reactivity that fuel or sustain these negative emotions [11,12]. In MBPs, emotional nonreactivity is specifically thought to occur through “decentering, in which the MBP participant is trained to attend to thoughts and feelings as mental events by noticing how they come and go in the mind” [13, p. 994]. Thus, MBP creators consider the approach orientation and decentering-based nonreactivity as an essential mechanism of MBP efficacy.

Importantly, although FA and OM are integrated within MBPs, each practice has different neural underpinnings and different cognitive, affective and behavioral consequences [7,14,15]. Furthermore, while it is assumed that combining FA and OM maximizes clinical benefit compared to either practice by itself [5], this assumption has never been empirically tested. In fact, in both Asian Buddhism and western science, the relative importance of FA and OM for the alleviation of affective disturbance has been debated [4,16]. However, because no previous studies have compared single-ingredient FA and OM training programs in meditation-naïve participants, the contribution of each practice to therapeutic outcomes is unknown. In the treatment development part of the current trial, Britton et al. [17] created separate, validated, single-ingredient training programs for FA and OM, providing a way to test the individual contributions of each component.

The current trial was modeled after the classic dismantling design from Jacobson et al. (1996), in which cognitive behavioral therapy (CBT) was dismantled into its separate cognitive and behavioral components, which were compared to the combination (CBT) in a three-armed randomized controlled trial. The current analysis assessed the unique effects of FA and OM on different forms of affective disturbance and evaluated whether the combination of FA and OM (MBCT) is more effective compared to either component by itself. In line with recent recommendations [18–20], this project used a multi-method approach to assess comparative efficacy in multiple dimensions of affective disturbance, assessed by both observer-rated and self-report methods, across multiple time frames (during-treatment, post-treatment, long-term), and in terms of statistical as well as clinical significance. Primary outcomes included stress, anxiety, and depression, which represent non-redundant dimensions of affective disturbance and which are the most frequent reasons for using mindfulness meditation [1]. In addition to immediate post-treatment effects, the trial assessed the rapidity of response by assessing differential treatment effects *during* treatment, not only because faster treatments are more efficient and require less time and burden, but also because rapid response or “early gains” during treatment are consistent predictors of better short- and long-term outcomes and less attrition [21–23]. Similarly, long-term maintenance of treatment-related improvements is an essential dimension of efficacy, as treatments that maintain gains are clearly superior to those that lose them. Finally, since treatment efficacy is also based on the ratio of benefits and harms, the trial describes outcomes in terms of both clinically meaningful improvements and deteriorations [24].

## Methods

### Participants

The sample was intended to be representative of Americans seeking meditation training, who typically exhibit clinical, sub-clinical and transdiagnostic expressions of affective disturbances, including anxiety, depression and stress [1]. Inclusion criteria were: age 18–65 years, English-speaking, mild-severe levels of depression (Inventory of Depressive Symptomatology [IDS] score of 10–48) and persistent negative affect (Positive and Negative Affect Schedule [PANAS] negative affect scale score > 18). Exclusion criteria were: extremely severe depression (IDS > 48); active suicidal ideation; history of bipolar, psychotic, borderline or antisocial personality disorders; repeated self-harm or organic brain damage; current panic, post-traumatic stress disorder, obsessive-compulsive disorder, eating disorder or substance abuse; current psychotherapy; a regular meditation practice or any change in antidepressant medication in the last eight weeks. See Britton et al. [17] for details.

### Setting and oversight

The trial took place at Brown University from November 2012 to March 2016, was approved by the Brown University Institutional Review Board (#1105000399), registered with clinicaltrials.gov (NCT01831362), and supervised by an independent Data Safety Monitoring Board and NCCIH’s Office of Clinical and Regulatory Affairs (OCRA). A study protocol was reviewed and approved by OCRA prior to study enrollment.

### Sample size and sampling

Since differences in clinical outcomes (depression, anxiety) between active treatments tend to be small [25], we planned for a sample size of 90 (30 per treatment) which would be able to detect a small effect size (*d* = 0.34, power > .80, *α* = 0.05, two-tailed). Based on prior studies [26], we estimated a 15% attrition rate, and thus needed to enroll a total of 105 subjects (35 per group) in order to have 90 completers. Participants were recruited through community advertisements describing meditation for stress, anxiety and depression. Eligible participants provided written, informed consent, and did not receive financial compensation.

### Randomization and design

The trial was a 3-armed cluster-randomized trial with a 1:1:1 allocation ratio without stratification by baseline variables (which would be added as covariates if groups differ). The allocation sequence was generated by an independent statistician, using a specified seed and a Latin square design that allocated to nine separate treatment cycles (three of each type of treatment type) with 10–12 participants each. The results of each treatment randomization were recorded and communicated to participants by an independent research assistant that was not involved in assessments. Because all three active treatments were presented as “mindfulness training,” participants were unaware of alternative treatments. Baseline assessment occurred before randomization and outcomes assessors were blind to treatment allocation. The PI, who was also a co-therapist for each treatment provided de-identified codes to signify different treatments during analysis.

### Interventions

#### Mindfulness-based cognitive therapy (MBCT)

The MBCT treatment [5] combined the principles and format of MBSR [27] with elements of CBT [28], in a client-centered group-based intervention. The meditation techniques used in MBCT contain a combination of both FA and OM meditations, which are described above (see Introduction).

#### Focused attention (FA) program

The main FA practices introduced participants to six possible anchors on which to focus their attention: three breath placements (nostrils, chest or belly), hands, feet and sound, with an additional set of optional anchors added at the sixth week. Participants were encouraged to choose at least two anchors as a primary and secondary object of meditation.

#### Open monitoring (OM) program

OM exercises began with mentally noting and labeling thoughts, emotions and sensations according to their phenomenological classification (e.g. sound, touch, thought, etc.) and valence (e.g. positive, negative, or neutral), ultimately transitioning to silent noticing in more advanced stages of practice. Participants were encouraged to notice biases in attentional allocation and to apply “balanced coverage” across different phenomenological categories.

### Treatment validation and fidelity

Britton et al. [17] describes in detail the creation and validation of two separate 8-week FA and OM meditation training programs that are structurally equivalent to MBCT, including session-by-session descriptions and transcripts of meditation practices. Briefly, all treatments were matched on participant-level variables (sample characteristics), treatment-level variables (program structure and duration, program materials, class size, attendance, homework adherence, etc.) and instructor-level variables (past meditation/clinical training, participant ratings and adherence/fidelity). Classes met for three hours once per week for eight weeks with a full-day silent retreat and formal practice homework consisting of 45 minutes per day, six days per week. Participants received basic training in targeted practices (FA, OM or combination) during weeks 1–4 and then learned how to apply these practices to regulate negative emotions (i.e., “working with difficulties”) in weeks 5–8.

The treatments were also confirmed to be differentially valid or differ in terms of program materials (handouts, audiotapes and readers) and differential mechanistic target engagement (skills acquired) as predicted by *a priori* hypotheses. In terms of program materials, FA program materials had more references to “targets,” “objects” or “anchors” of directed attention, while OM materials emphasized “tracking,” “noting” or “labeling” transient stimuli. In terms of participant skill acquisition, FA training resulted in the largest increase in attentional control, while OM training resulted in the greatest increase in emotional non-reactivity and decentering [17]. In addition, when encountering a negative thought or emotion, OM participants were more likely to attend *toward* the difficulty by nonjudgmentally observing, naming or labeling it. In contrast, FA participants were more likely to shift attention *away* from the difficulty by attending to their breath or another neutral object. Treatment fidelity was measured by independent raters of session audio tapes using standard and FA/OM-adapted versions of the MBCT adherence scale [29]. Adherence to treatment manuals was > 85% for all treatments (see Table 1 and Britton et al. [17] for details on adherence, fidelity, instructors and treatment validation).

**Table 1:** Sample characteristics.

	OM *n* = 36	MBCT *n* = 32	FA *n* = 36	Total *n* = 104
Female, *n* (*%*)	26 (72.2)	23 (71.9)	27 (75.0)	76 (73.1)
Age, *M* (*SD*)	40.0 (13.2)	38.6 (12.4)	42.1 (12.8)	40.3 (12.8)
AD meds, *n* (*%*)	12 (33.3)	12 (37.5)	11 (30.6)	35 (33.7)
Race, *n* (*%*)				
White	35 (97.2)	31 (96.8)	36 (100)	102 (98.0)
Asian	0 (0)	1 (3.2)	0 (0)	1 (1.0)
Not reported	1 (2.8)	0 (0)	0 (0)	1 (1.0)
Ethnicity, *n* (*%*)				
Hispanic/Latino	4 (11.1)	2 (6.5)	1 (2.8)	7 (6.8)
Non-Hispanic/Latino	32 (88.9)	30 (93.5)	35 (97.2)	97 (93.2)
Highest level of education, *n* (*%*)				
High school	1 (2.8)	2 (6.3)	0 (0)	3 (2.9)
College	16 (44.4)	18 (56.3)	22 (61.1)	56 (53.8)
Graduate	19 (52.8)	12 (37.5)	14 (38.9)	45 (43.3)
Axis I Diagnoses, *n* (%)				
Current clinical MDD	15 (41.7)	12 (37.5)	14 (38.9)	41 (39.4)
Current clinical GAD	18 (50.0)	14 (43.8)	20 (55.6)	52 (50.0)
Participant Adherence				
Total randomized, *n*	36	32	36	104
Completed intervention, *n* (*%*)^a^	31 (91.2)	30 (93.8)	35 (97.2)	96 (94.1)
Classes attended, *M* (*SD*)^b^	7.7 (1.8)	7.7 (1.8)	8.1 (1.3)	7.8 (1.6)
Meditation Homework Compliance				
8wk formal min/wk, *M* (*SD*)^b^	215.9 (72.2)	185.2 (82.6)	205.6 (65.9)	202.6 (73.7)
3mo formal min/wk, *M* (*SD*)^b^	104.2 (106.9)	97.8 (97.2)	100.0 (96.6)	100.7 (99.2)
Instructors Gender ratio (male: female) Combined meditation experience (years) # Clinical degrees # Ph.D.’s # MBSR/MBCT instructors	1:1 40 1 2 1	1:1 40 1 2 2	1:1 40 2 1 2	
Treatment Fidelity (*%*)	88.9	93.9	97.1	93.3

*Note*. AD meds = Antidepressant medication; MDD = Major Depressive Disorder; GAD = Generalized Anxiety Disorder; 8wk = during 8-week intervention; 3mo = period between week 8 and 3-month follow-up (week 20); # = “number of”.

^a^Completers only include participants who began treatment (i.e., attended the first class). Two additional participants dropped from OM before the beginning of treatment.

^b^These variables only include participants who completed all 8 weeks of treatment (FA, MBCT, OM *n’s* = 35, 30, 31, respectively).

### Measures

#### The Inventory of Depressive Symptomatology (IDS)

The IDS-C [30] is a 30-item clinician-administered interview designed to measure symptoms of unipolar major depression in the last week according to DSM-IV criteria. The IDS-C was administered at baseline, post-treatment (week 8) and 3-month follow-up (week 20) by graduate-level research assistants who were trained by and met high inter-rater reliability (Cohen’s kappa) with PhD-level clinicians (baseline, week 8, week 20 κs = 0.89, 0.93, 0.94, respectively).

#### The Depression Anxiety Stress Scales (DASS)

The DASS [31] is a 42-item self-report questionnaire that assesses anxiety, depression and stress symptoms in the last week. The DASS was administered at five timepoints: baseline, weeks 2, 4, 6, 8 and 20. The DASS subscales scales have been shown to have high convergent validity with other measures of stress, anxiety and depression and internal consistency in both clinical and nonclinical samples [31–33]. In the current study, DASS subscale reliabilities (Cronbach’s alpha) were as follows: depression (αs = 0.93–0.95), anxiety (αs = 0.77–0.85) and stress (αs = 0.88–0.92).

### Statistical analyses

All analyses were conducted using SAS software (version 9.4; SAS Institute, Cary, NC). Significance level was set at *p* < .05 (two-tailed) for all statistical tests. Standardized effect sizes were reported as Cohen’s *d* and interpreted in the following manner: small = 0.20, medium = 0.50 and large = 0.80 [34]. The unstandardized regression coefficient estimate (*b)*, which represents the real-world point changes in the specific instrument used, is provided in S1 and S2 Tables. Because current statistical reporting guidelines [35–38] recommend interpreting results according to effect size and not solely on statistical significance testing or dichotomous *p* value cut-offs, non-significant, trend-level (*p* ≥ .05 - .10) differences with an effect size of *d* > 0.30 are interpreted and discussed as meaningful [39].

#### Preliminary statistical analysis

Descriptive statistics for each treatment group were calculated, including participant characteristics (demographics, baseline psychopathology), participant engagement (attrition/attendance, meditation homework adherence) and treatment fidelity (Table 1).

#### Primary statistical analysis

Because the trial contained three active treatments and no minimal intervention or placebo condition, within-group changes in the outcome measures over time could be due to non-specific effects such as regression to the mean or passage of time. Thus, the primary analysis focused on between-group comparisons to investigate differential treatment effects. Within-group Cohen’s *d* effect sizes from baseline to post-treatment (week 8) and 3-month follow up (week 20) are also included in S2 Table in order to provide effect size comparisons with other trials and to aid interpretation of the between-group tests. Within-group effect sizes were calculated by dividing the *t*-value from a paired samples *t*-test comparing scores at baseline to week 8 and week 20, respectively, by the square root of the number of non-missing observations across the two timepoints [40,41].

Using an intent-to-treat (ITT) approach that included all randomized participants (*n* = 104), regardless of missing outcomes or adherence to protocol, mixed effects analysis of covariance models were used to identify differences between groups at week 8 and week 20 for each clinical outcome, adjusting for baseline levels of each outcome. Each model included the group, timepoint, group x timepoint interaction and baseline score as fixed effects independent variables and random subject effects to account for the correlation among the repeated measures obtained from the same participant. We fit both homogeneous and heterogeneous variance models. The former assumed the same covariance parameters of the repeated measures for the three treatments, while the latter allowed for different covariance parameters. The homogeneous variance model had a better fit to the data based on the Bayesian Information Criterion (BIC). The regression coefficients of the fixed effects independent variables estimated from the mixed effects ANCOVA model were then used to derive the estimates of the mean differences between groups for each outcome measure at each timepoint and the corresponding significance level (*p* value). To account for multiple comparisons, all *p* values for primary analyses were adjusted using a false discovery rate (FDR) procedure implemented in SAS [42]. Between-group Cohen’s *d* effect sizes were calculated by dividing the estimated difference in means between groups from the mixed effects ANCOVA model by the standard deviation of the estimated mean difference.

The analysis plan included in the trial registration specifies that models would also include meditation practice amount, age, sex and education variables as covariates but these were ultimately omitted in order to specify more parsimonious models. Additionally, while the registered analysis plan includes well-being as a primary outcome, clinical cutoffs indicating clinical significance were not available for the Well-being Scale. Consequently, well-being results will be presented in a separate manuscript in order to focus on clinically significant changes in affective disturbance and to maintain parallel structure between primary and exploratory analyses (see below).

#### Exploratory statistical analyses

To assess rapidity of response, within-group effects of DASS stress, anxiety, and depression during the treatment are reported as “first significant improvements” (FSI). FSI indicates statistically significant within-group differences from baseline in variables assessed bi-weekly (i.e., at weeks 2, 4, 6, 8) during treatment.

Clinically meaningful change was calculated using the Reliable Change Index (RCI) [24,43] of each outcome at each timepoint. Change scores from baseline to each timepoint were classified into three categories: reliable improvement, reliable deterioration or no reliable change. S1 Appendix contains information about the RCI analysis.

## Results

### Preliminary results

**Fig 1:**
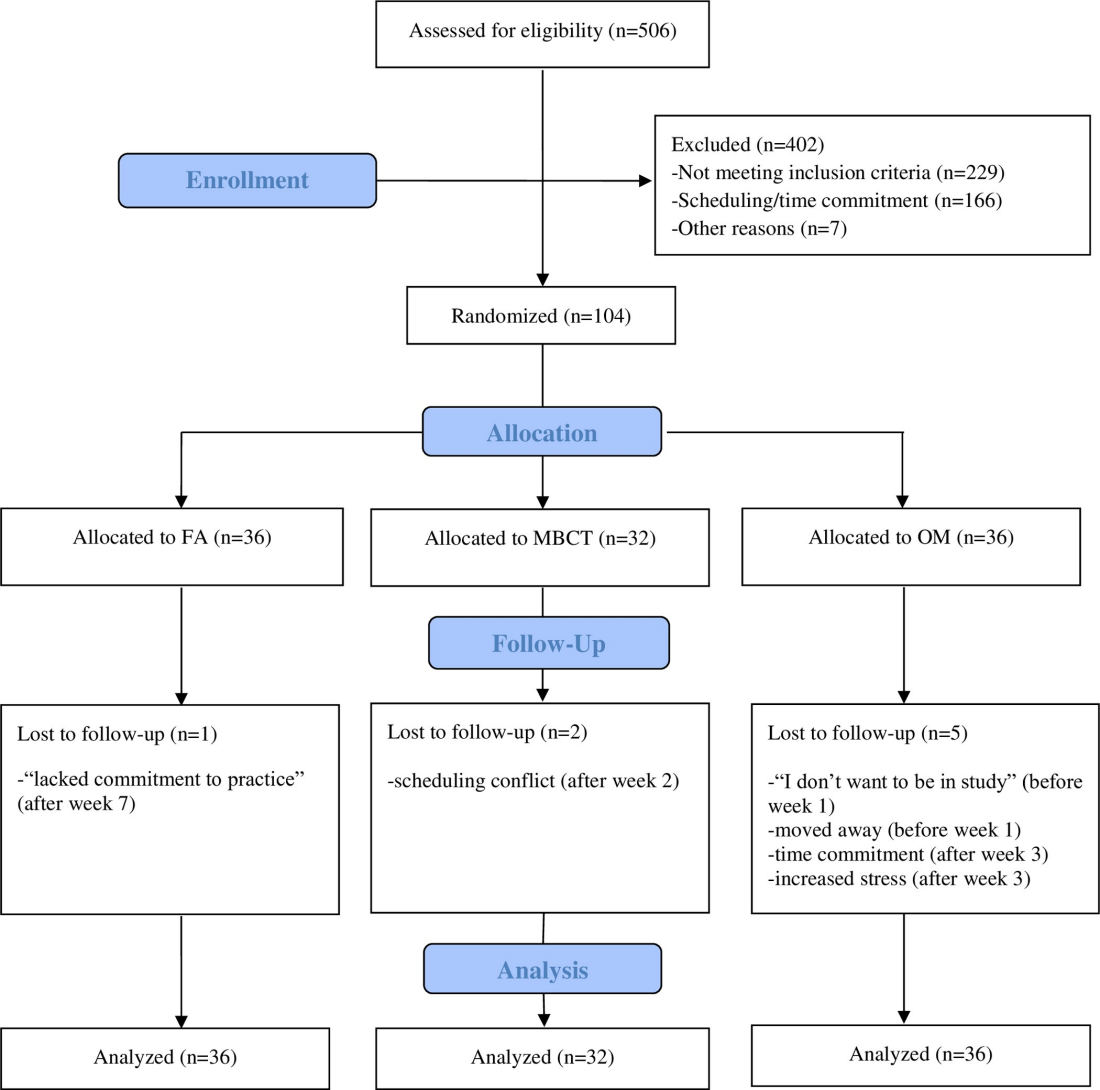
Participant flow. FA = Focused attention; MBCT = Mindfulness-based cognitive therapy; OM = Open monitoring.

Fig 1 describes participant flow, attrition and reasons for dropout. Table 1 describes participant flow, sample and instructor characteristics, and treatment adherence and fidelity. As described in Table 1, with additional information provided in Britton et al. [17], all three treatments were comparable in terms of participant demographics (age, gender, education, race/ethnicity), antidepressant use, diagnoses of Axis I disorders, baseline symptom severity (IDS, DASS depression and stress scores), participant attrition, attendance and meditation homework compliance (both during and post-intervention), intervention instructors (e.g., gender ratio, prior years of meditation experience), and instructor treatment fidelity. Although baseline DASS anxiety scores were significantly higher in the OM group (*p* = .04) this was accounted for by baseline-adjusted estimates for between-group comparisons resulting from the mixed effects ANCOVA models.

### Primary results

**Fig 2:**
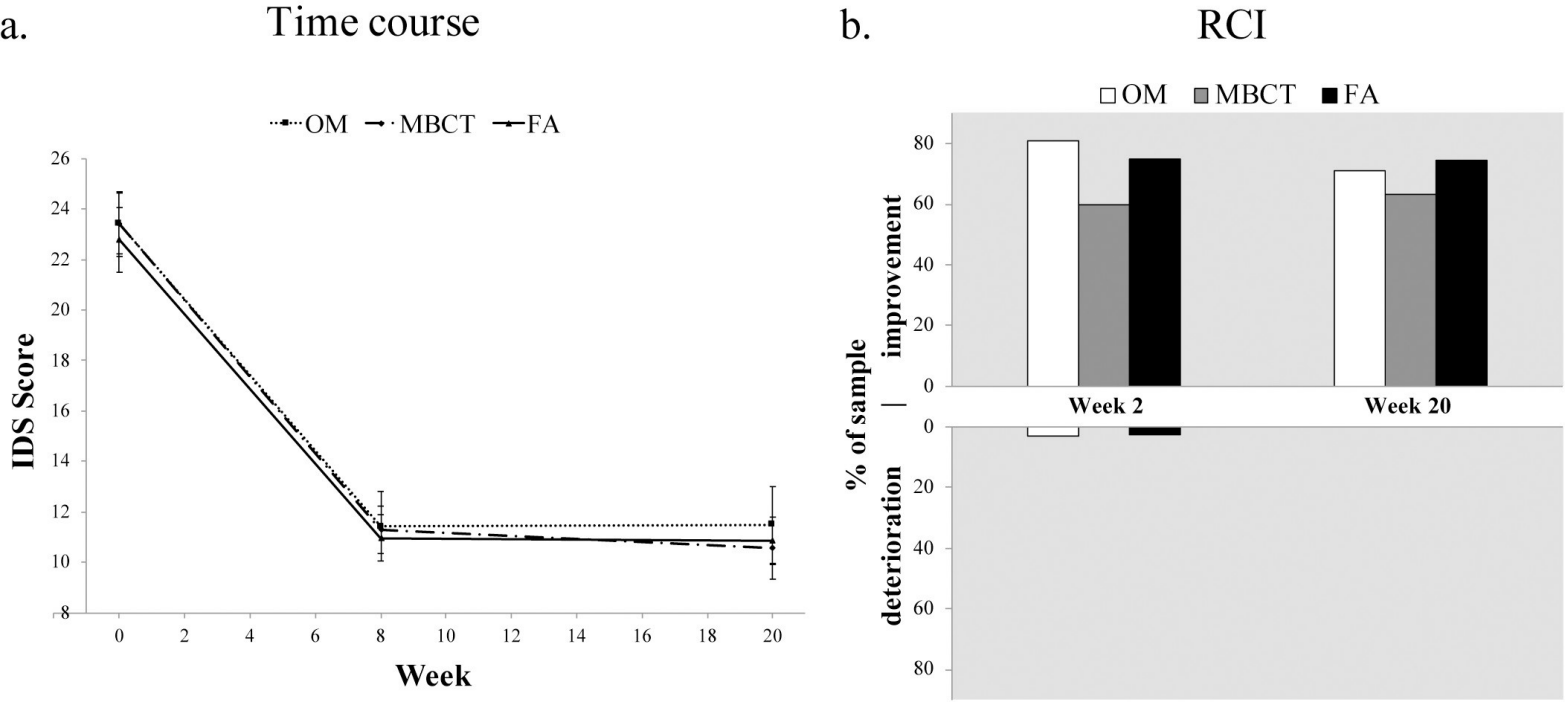
Differential treatment effects and clinically significant change in depression (IDS) for all three treatments over time. (a) Group means (symbol) and standard error (error bars) at baseline (week 0), post-treatment (week 8) and follow-up (week 20) in intent-to-treat regression analysis (*n* = 104). (b) Reliable change index (RCI) at each timepoint relative to baseline scores. Upward and downward bars signify % of each treatment sample showing clinically significant improvements and deteriorations, respectively. Percent of sample with no reliable change is not shown (see S1 Appendix).

**Fig 3:**
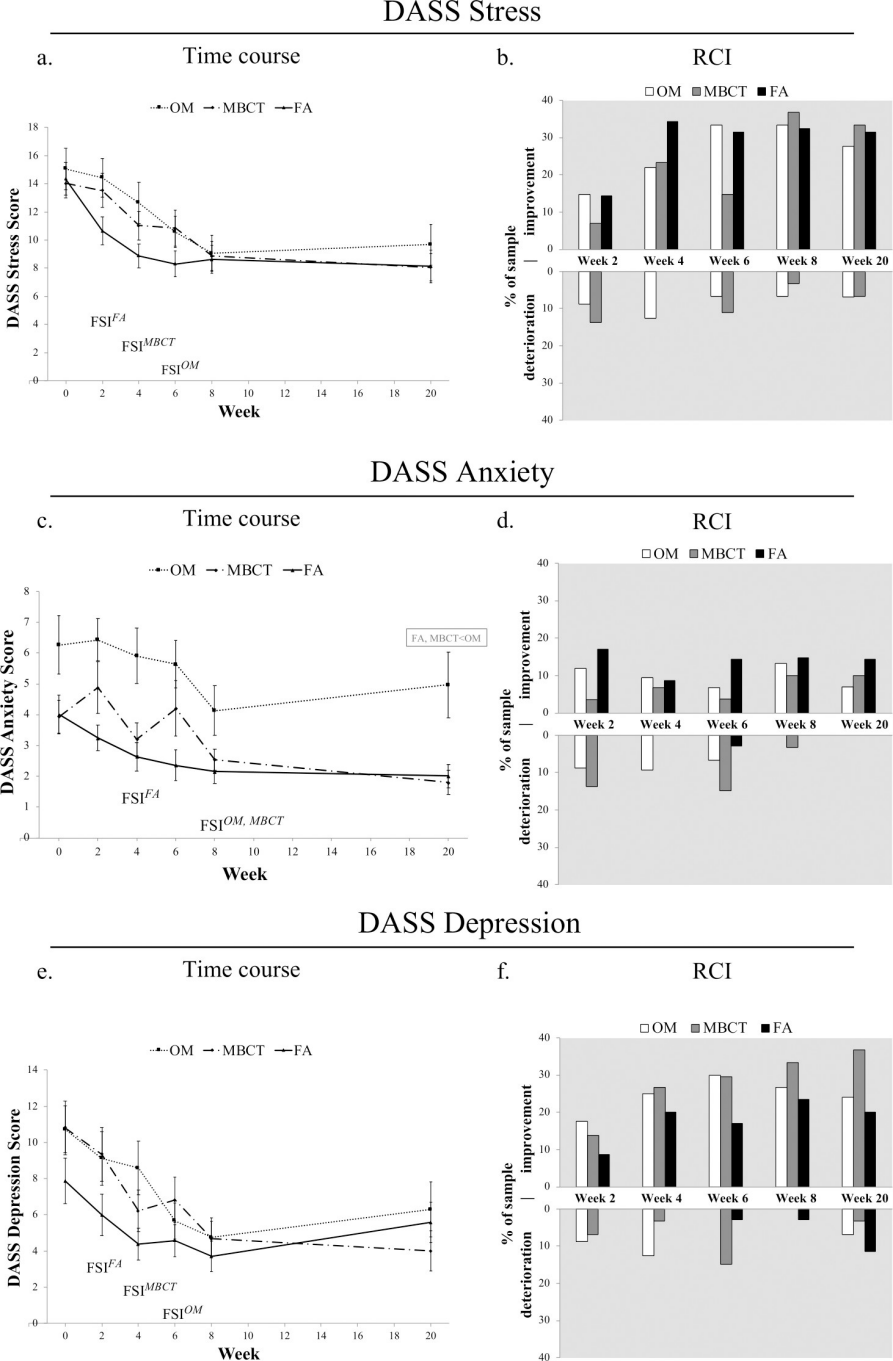
Differential treatment effects and clinically significant changes in stress, anxiety and depression (DASS). (a,c,e) Group means (symbol) and standard error (error bars) at weeks 0, 2, 4, 6, 8 and 20 in intent-to-treat regression analysis (*n* = 104). Gray text boxes denote statistically meaningful between-group differences (*d* > 0.30). (b,d,f) Reliable change index (RCI) at each timepoint relative to baseline scores. Upward and downward bars signify % of each treatment sample showing clinically significant improvements and deteriorations, respectively. Percent of sample with no reliable change is not shown (see S1 Appendix).

Figs 2 and 3 and S1 Table display differential treatment effects (between-group comparisons) for all measures. Group means did not significantly or meaningfully differ at week 8 or week 20 for depression (IDS; see Fig 2A; and DASS; see Fig 3E), and stress (DASS; see Fig 3A). For anxiety (DASS), while no significant or meaningful differences between groups were found at week 8, anxiety was lower in both FA and MBCT compared to OM at week 20 (*d*s = 0.36, 0.40 respectively; see Fig 3C and S1 Table).

To contextualize between-treatment differences within overall efficacy, all treatments produced significant medium to large effect sizes (*ds* = 0.42–1.65) for all outcomes with two exceptions of non-significant small effect sizes at week 20 for OM anxiety and FA depression (*d*s = 0.27 and 0.30, respectively). See S2 Table for details.

### Exploratory results

#### Depression (IDS)

Clinically meaningful changes paralleled statistical analysis. A large percentage of participants in all three treatments (60–81%) had reliable improvements from baseline to week 8 that were maintained at week 20 (Fig 2B). OM showed the largest percentage of reliable improvements from baseline to week 8 (81%), followed by FA (75%) and then MBCT (60%). Very few reliable deteriorations from baseline to week 8 were found: 2.8% for FA, 3.2% for OM, none for MBCT. See S1 Appendix (Table S4.2).

#### Stress (DASS)

**Fig 4:**
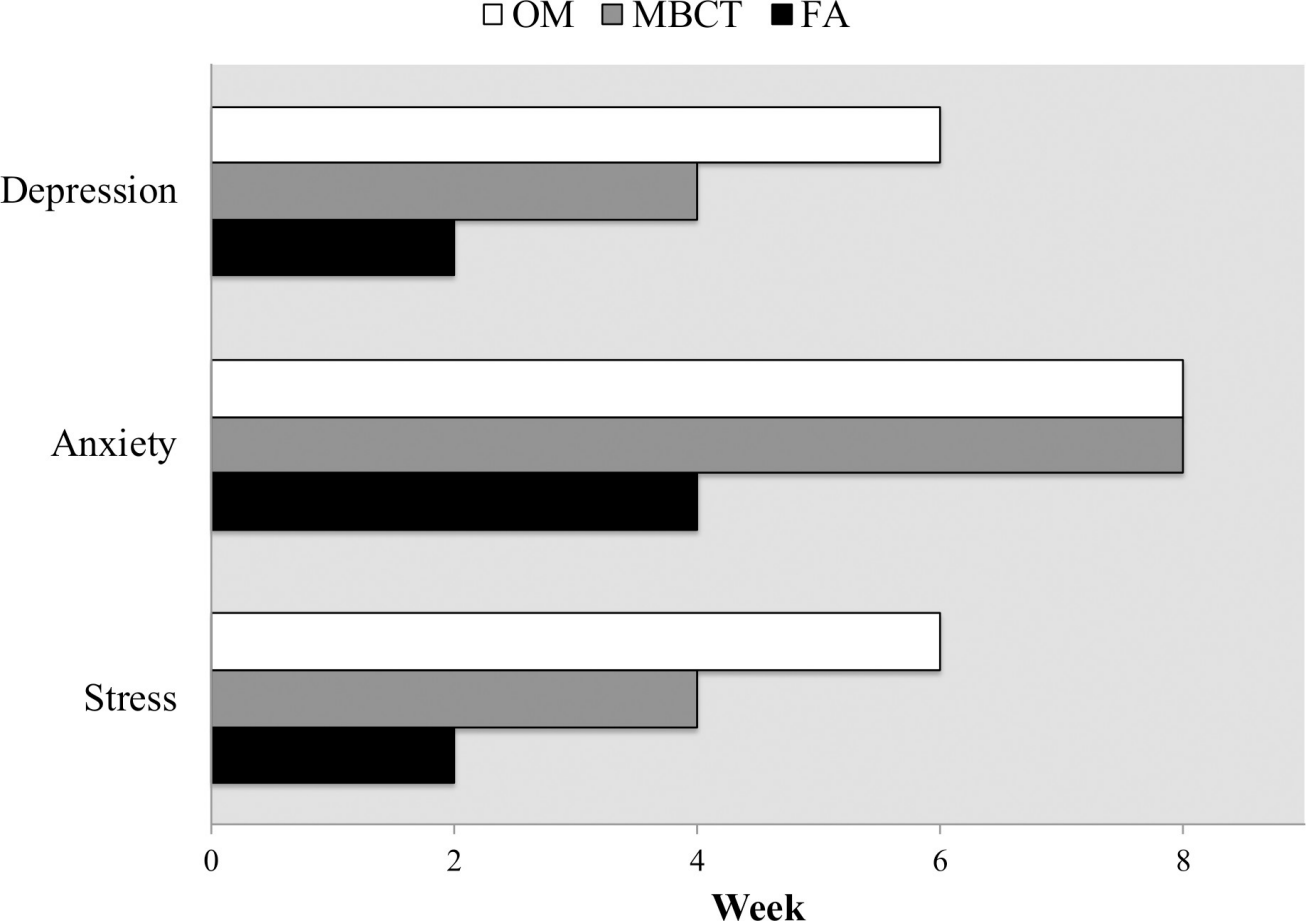
Number of weeks until first significant improvement (FSI) from baseline in stress, anxiety and depression (DASS) during the 8-week intervention.

First significant improvement occurred at week 2 for FA, (*d* = 0.59, *p* < .001), week 4 for MBCT (*d =* 0.53, *p* = .012) and week 6 for OM (*d* = 0.60, *p* < .001). See Figs 3A and 4. More than one-third (32–37%) of participants in all three treatments had reliable and clinically significant improvements in self-reported stress from baseline to week 8, which were largely maintained at week 20 (see Fig 3B). FA yielded these effects earliest at week 4, OM at week 6 and MBCT at week 8. Reliable deteriorations in terms of clinically significant increases in stress were absent in the FA group at all timepoints but occurred in OM at all five timepoints and in MBCT at four of five timepoints, including > 5% at week 20, signifying lasting increases in stress post-MBP. See Fig 3B and S1 Appendix (Table S4.3).

#### Anxiety (DASS)

First significant improvement in anxiety occurred at week 4 for FA (*d* = 0.32, *p* = .036) and week 8 for both MBCT (*d* = 0.56, *p* = .044) and OM (*d* = 0.42, *p* = .002). See Figs 3C and 4. Reliable improvements in self-reported anxiety were found in 10–15% of participants in all treatments by week 8 (see Fig 3D). FA showed a pattern of the most rapid and reliable improvements in anxiety, with the fewest cases of deterioration (3% at week 6). MBCT tended to show the slowest and least reliable improvements and the highest rates of deterioration. For example, increases in anxiety were almost three times more likely than decreases in anxiety at weeks 2 and 6 in MBCT, with nearly 15% exhibiting clinically significant increases (i.e., shifting from normal to moderate or severe anxiety). Reliable improvements and deteriorations in anxiety were about equally as likely at weeks 2, 4 and 6 in OM. No treatment produced lasting increases in anxiety at week 20, although more than 80% of participants in each treatment also failed to show any clinically significant change in anxiety at any timepoint. See S1 Appendix (Table S4.3) for details.

#### Depression (DASS)

First significant improvement occurred at week 2 for FA (*d* = 0.27, *p* = .0449), week 4 for MBCT (*d* = 0.62, *p* = .001), week 6 for OM (*d* = 0.76, *p* < .001). See Figs 3E and 4. Reliable improvements in depression were found in one-third of MBCT participants (33%), and roughly one-quarter in FA (24%) and OM (27%) by week 8 (see Fig 3F). At week 20, reliable improvement had increased to 37% in MBCT but decreased to 20% and 24% for FA and OM, respectively. MBCT showed the highest and most sustainable rate of reliable improvement, but also the highest rate of reliable deterioration (15% at week 6). OM closely paralleled MBCT but had slightly lower rates of reliable improvement as well as deterioration. FA showed the lowest level of reliable improvements and deteriorations during treatment, but the highest level of deterioration from baseline to week 20 (11.4%) indicating long-term, clinically significant increases in depression. See Fig 3F and S1 Appendix (Table S4.3).

## Discussion

This study followed Jacobson et al.’s [44] classic dismantling design to compare the independent contributions of FA and OM with their combined form (standard MBCT) in terms of addressing affective disturbances typical of Americans who use MBPs. The current analysis investigated differential treatment effects and patterns of reliable improvement and deterioration for depression, anxiety, and stress during and following eight weeks of treatment. While all treatments were largely comparable in their effects at post-treatment (week 8), the treatments differed in their long-term effects, rapidity of response and pattern of deteriorations. Each result will be summarized below, followed by a discussion of the potential reasons and implications of each type of difference.

### Post-treatment effects

When assessed immediately post-treatment (week 8), all three treatments showed similar effects on all outcomes, with medium to large effects on both clinician-rated and self-reported depression, and stress, and small to medium effects on anxiety.

### Long-term effects

At the week 20 follow-up, while treatments were largely comparable for stress, they diverged most clearly for anxiety. MBCT and FA maintained their medium to large treatment-related gains, while OM’s gains were lost and anxiety scores returned to pre-treatment levels.

Similarly, the lowest levels of reliable improvement at week 20 were found in anxiety in the OM group.

Results for depression varied by both measurement and mode of analysis, with clinician-rated depression suggesting treatment parity, but self-reported depression suggesting an advantage for MBCT over FA, which lost its treatment-related gains at week 20. Similarly, RCI analysis found the highest rate of reliable improvement and lowest rate of deterioration for MBCT while FA showed the inverse, the lowest rate of improvement and highest deteriorations.

### Rapidity of response

Across self-reported stress, anxiety and depression, statistically significant improvements occurred soonest for FA, followed by MBCT, and with OM consistently exhibiting the slowest rates of improvement. Significant improvements in depression and stress occurred twice as fast in FA compared to MBCT and three times as fast as OM. Similarly, significant improvements in anxiety occurred twice as fast in FA compared to both MBCT and OM. RCI analysis paralleled statistical findings for anxiety and stress but not depression: FA showed the largest and fastest reliable improvements and minimal deteriorations in anxiety and stress, while reliable improvements in depression scores lagged behind both MBCT and OM.

### Deterioration profiles

Clinically meaningful deteriorations differed across treatment, outcome and timepoint. Both MBCT and OM showed reliable deteriorations in all self-reported outcomes (depression, anxiety and stress) at nearly every treatment timepoint, with long-term, lasting deteriorations in stress and depression. Consistent with findings that early deteriorations predict attrition [22], one OM participant dropped out after class 3 because of “increased stress.” In contrast, FA showed minimal deteriorations on any outcome during treatment, but long-term deteriorations in 11.4% of participants in self-reported depression. While anxiety deteriorations were common in MBCT and OM during treatment, long-term deteriorations were absent, although the vast majority of anxiety scores did not show reliable changes. Notably, nearly all deteriorations across all treatments occurred in individuals who were in the normal, non-clinical range at baseline, but developed clinically significant symptoms during treatment. See S1 Appendix for RCI plots for each treatment, outcome and timepoint.

### Clinical implications: Practice by condition matching

The results of the current study provide preliminary evidence-based recommendations to tailor MBPs and their components to the needs and goals of different conditions and individuals.

#### Stress

All three treatments showed equal and sustained medium to large improvements in stress, with similar levels of clinical significance at post-treatment and follow-up. However, FA produced significant improvement two to three times as fast as the other treatments, and early gains are associated with larger effect sizes and decreased attrition [22]. In addition, while MBCT and OM showed reliable increases in stress at multiple timepoints, including long-term, lasting deteriorations, FA did not result in stress-related deteriorations at any timepoint. Given that MBPs have not been particularly efficacious compared to other treatments for stress [45], increasing FA-related practices may be worth considering when tailoring MBPs for stress.

#### Anxiety

FA demonstrated superiority over the other two treatments on several indices of anxiety. FA showed statistically significant and clinically meaningful improvements that were rapid, sustained and accompanied by minimal deteriorations. FA resulted in statistically significant improvement without deteriorations by week 4. In contrast, both MBCT and OM were more likely to produce deteriorations than improvements in the first six weeks of treatment, which resulted in termination of treatment for at least one participant. Clinically significant improvements were highest in the FA group at all timepoints.

Treatment-related improvements in anxiety for OM were lost at week 20. Because baseline anxiety scores were significantly higher in the OM group than in the other treatments, it is difficult whether to interpret OM as contraindicated for anxiety in general and/or for higher levels of anxiety. Inspection of the RCI plots (S1 Appendix) supports both hypotheses. Individuals in the OM group who had high levels of anxiety at baseline but showed no improvement at week 20 largely accounted for the group differences and lack of effect for OM on anxiety, even though they had more room for improvement. RCI plots also show that some participants increased from non-clinical levels of anxiety at baseline to clinical levels during treatment in both OM and MBCT, but not FA.

It is important to note that, compared with other outcomes, none of the treatments were particularly efficacious at improving anxiety. Despite medium effect sizes, clinically significant improvements occurred in only 10–15% of participants, while 85–90% did not show any meaningful change. Lack of meaningful change may be related to average baseline DASS scores in the non-clinical range, although 40–50% of participants met diagnostic criteria for generalized anxiety disorder (GAD) in a clinical interview. Lack of efficacy for anxiety in MBPs is echoed by a recent meta-analysis [46], which found no benefit for and thus recommends against using MBPs for clinical levels of anxiety given that other more efficacious treatments are available. The current study adds nuance to these conclusions and suggests that the OM component of MBPs may be the source of contraindication, while the FA dimension may actually be beneficial for anxiety. Together, these findings suggest that increasing FA and/or decreasing OM may be potential ways to tailor MBPs to maximize their efficacy for anxiety.

#### Depression

There were no statistically significant differences between treatments on either clinician-rated or self-reported depression at any timepoint, which suggests that MBCT showed little advantage over either FA or OM alone. However, results differed both by measure and mode of analysis. The RCI analysis presented conflicting results for the different depression measures. For clinician-rated depression, the RCI analysis produced the least favorable percentage of reliable improvements for MBCT at both post-treatment and follow-up. For self-reported depression, MBCT had the most favorable ratio of lasting improvements compared to lasting deteriorations. Other studies’ rates of reliable improvement (15–25%) and reliable deterioration (3–5%) in MBCT [47,48] are more consistent with the current study’s self-reported depression data.

The pattern of results for depression in the FA group may also warrant more attention. FA showed rapid improvement in statistical but not clinical significance, which lagged behind MBCT and OM at all timepoints. In addition, reliable deteriorations in the FA group accumulated over time, with 11% showing lasting deteriorations at follow-up and a loss of treatment-related gains. This pattern suggests that FA may be ineffective for and/or cause increases in depression in some people when continued beyond optimal levels [49].

In contrast to stress and anxiety, for which FA was favored over MBCT, depression results favored MBCT over FA. Given that MBCT was specifically designed for and shown to be effective for preventing depressive relapse in chronic depression [5,50] and has also shown promise in improving acute depression [46], MBCT may remain the best choice for depression.

### Current results in the context of meditation and clinical treatment research

The results in the current study show parallels with the theory and practice of Buddhist meditation as well as with clinical approaches that aim to treat anxiety and depression through targeting attentional biases (e.g., exposure and attention bias modification). In line with mechanism-focused experimental medicine [49,51], both approaches predict that treatments will be maximally beneficial when they reverse baseline pathology or deficits and will be ineffective or contraindicated if baseline pathology is exacerbated or a new imbalance is created. The theory and practice of Buddhist meditation characterizes imbalances in terms of excessive “excitation” (or hyperarousal) and excessive “laxity” (or hypoarousal) [52]. Clinical treatments assess baseline deficits in terms of the tendency to under-engage (avoid) or over-engage (negative bias) with threat or negative emotions [7,53].

FA practice ostensibly calms or reduces hyperarousal and also directs attention away from negative emotions. These two mechanisms suggest that FA would be most beneficial to individuals with symptoms of hyperarousal (stress, anxiety) or who over-attend to threat. In clinical studies, training attention away from negative stimuli can reduce both depression and anxiety symptoms [54,55]. Conversely, both mechanisms would also suggest that FA would be least effective and potentially contraindicated for individuals with low levels of arousal and/or the tendency to avoid threat, or when baseline deficits are overcorrected. Buddhist meditation manuals caution that overuse of tranquility practices like FA can lead to excessive lethargy and “dullness,” where the meditator becomes “withdrawn, physically inactive and mentally depressed” [52, p. 507]. These predictions play out in the current study: FA appears to be maximally effective for anxiety and stress, states characterized by high levels of arousal and negative attentional biases [56]. Short-term practice of FA improved depression, but continued practice reversed gains and caused the development of clinically significant depression in previously non-depressed participants.

OM, which directs attention toward threat and increases excitation and arousal, was the least effective for and caused many deteriorations in stress and anxiety. In the long term, OM had no significant benefit for anxiety and exhibited significantly higher levels of anxiety compared to FA and MBCT at week 20. This finding mirrors Brake et al.’s [11] findings where short-term mindfulness inductions (“notice your thoughts without having to react to them”) were no more effective for anxiety disorders and were associated with higher levels of subjective distress compared to “focus[ing] your attention on something else” (p. 229). Similarly, training individuals with nonclinical levels of anxiety and/or low levels of avoidance to attend toward threat (i.e. exposure) has been previously reported to be both ineffective and increase anxiety [53,57]. These findings are also anticipated in the theory and practice of Buddhist meditation. When the increased “phenomenal or subjective intensity” cultivated in OM is not counterbalanced with the calm and stability of tranquility practices like FA, “an excess of physical and mental tension may develop” [52, p. 507].

The current study challenged at least two prevailing assumptions about MBCT, namely, that MBCT would be superior to either FA or OM component alone, and that the approach orientation (attention toward rather than away from difficulty) is central to MBCT’s efficacy across all conditions. We previously reported that, as predicted, MBCT and OM produced the highest level of decentering and nonreactivity and directed attention *toward* difficulty, while FA trained attention away from difficulty [17]. However, neither MBCT nor OM clearly outperformed FA on any measure or timepoint. On average, across all timepoints and measures, MBCT and OM had the higher average rate of deterioration (5.6% and 5.4%, respectively) than FA (1.2%). Thus, MBCT and OM were more than 4 times more likely to produce deteriorations at any given timepoint compared to FA.

Despite these challenges, there are still reasons to believe the combination (MBCT) may have advantages. As seen in the current study, FA without OM can contribute to increases in depression, while OM without FA can increase stress and anxiety. Individuals with depression and anxiety can have biases toward or away from threat [56,57], and therefore a treatment that could correct both types of biases (i.e. includes FA and OM) would be the most efficacious for the most people. However, offering both FA and OM could also increase the likelihood of exacerbating baseline deficits, so it might also be expected to produce deteriorations, which was indeed the case for MBCT in this study. In order to maximize efficacy and minimize deteriorations, MBCT would need embrace its multi-practice, multi-mechanism identity and teach participants how to optimally match their conditions with the most appropriate practice, rather than universally encouraging individuals to turn towards difficulty [49].

### Limitations and future directions

Given that this is one of the first studies to compare FA, OM and MBCT in a randomized controlled trial, the results of this study should be considered preliminary until replicated. Additional limitations that qualify the above conclusions are listed below along with recommendations for future studies.

#### Design

The sample size and choice of comparison group may limit conclusions. The absence of a non-meditation group limits the ability to conclude that the observed improvements were related to meditation training rather than non-specific factors (e.g. the passage of time, group support, etc.). However, the efficacy of MBPs for anxiety, stress, and depression, compared to no-treatment controls has been repeatedly demonstrated [58,59], and the pre-post effect size of all three treatments was similar to other MBPs studies [60]. Observed between-group differences in this study, i.e., higher anxiety in OM at week 20 compared to FA and MBCT, should be confirmed in follow-up replication studies with larger sample sizes and greater statistical power.

#### Treatment differences

The original trial registration specified that demographic variables such as age, sex and education would be included as covariates in statistical models given that these were considered variables that could have a potential impact on treatment outcomes. However, these variables were ultimately omitted in order to specify more parsimonious models, and randomization was successful in creating similarity across treatments for these and most other variables at baseline. However, differences in self-reported anxiety and teacher ratings may complicate conclusions. Although frequency of anxiety disorders (GAD) was similar across treatments, self-reported anxiety symptoms were higher in OM and were addressed with baseline adjustment. While instructors were comparable for years of meditation experience, gender ratio, treatment adherence/fidelity and overall treatment ratings, the FA instructors received more positive ratings on both empathy and working alliance, which could have contributed to FA’s accelerated improvements [17]. While one of the instructors was the same across all treatments, the FA-specific instructor was the only full-time clinician and had more clinical experience than the other instructors. While neither clinical experience nor mindfulness instructor competence have been clearly linked to outcomes [61,62], other instructor qualities such as empathy and therapeutic relationship quality have been linked to better outcomes [63]. Since the instructors were rated after treatment was completed, it is difficult to interpret whether better ratings in FA were a cause or an effect of successful treatment. However, while it may be difficult to differentiate the influence of the meditation practice and the instructor, the current study identifies and emphasizes both as important factors that could impact treatment outcomes.

#### Measurement effects

Efficacy varied by type of measurement, including time frame, statistical vs. clinical significance, and clinician-rated vs. self-report methods. Similar to other studies, clinician-rated depression generated larger effect sizes than self-reported depression suggesting “either self-report measures are more conservative or that clinician-rated improvement is more sensitive to change” [64, p. 772]. Since participants are more likely to disclose sensitive information such as depression symptoms and negative treatment effects in self-reports than face-to-face interviews [65], researchers have been encouraged to use a combination of clinician-based and self-rated tools [18]. Because treatment selection decisions are determined by multiple factors beyond statistically significant pre-post treatment change, triangulation of multiple types of measurements yield the most reliable estimates [18]. Furthermore, deterioration in the target variables measured in the current manuscript is only a partial assessment of harms [66]. Systematic assessment of the emergence of novel symptoms, of known or expected treatment-specific effects, as well as unexpected side effects will be addressed in a separate manuscript [49].

#### Length of follow-up

Though a 3-month follow-up was administered to assess treatment differences in the ability to retain improvements made during the 8-week treatment, it is unknown how the treatments would have compared at six months, one year, or multiple years later. Potential differences among the three interventions might have become more apparent over longer periods of time.

#### Differential mechanistic mediation

FA and OM successfully cultivated differential skills as intended [17]. However, it remains to be tested whether practice-specific skills acquired during the course of the intervention differentially mediated outcomes for these two treatments or if improvements were instead related to non-specific factors [20].

#### Order effects

The inclusion of standard MBCT where FA training precedes OM training precludes any conclusions about an order effect until the opposite order (OM, FA) is also included as a comparison. Although MBCT does not neatly delineate FA and OM style practices, its sequencing of practices across sessions is consistent with certain approaches to Buddhist meditation in which a practitioner entrains in FA before engaging with OM style practices [5]. However, looking broadly across Buddhist traditions, it is possible to find support for exclusively FA paths of contemplative development, for FA leading to OM style practice, for exclusively OM style practices, and for approaches that integrate FA and OM [16,52,67]. In the context of the clinical psychology of MBPs, the optimal combination, balance and sequencing of these two practices should be explored through further empirical research.

#### Generalizability

Several dimensions of the current study limit the generalizability of its findings. The sample was both self-selected and carefully screened according to standard MBP exclusion criteria [8,68]. Therefore, these findings may not extend to individuals not seeking meditation, to children or the elderly, to those with other physical or mental health conditions, or those who do not undergo a lengthy individual screening process. Similarly, with more than 20 years of meditation experience and graduate level clinical, research and/or monastic training, the instructors in the current trial were likely more qualified than many MBP instructors, although MBP instructor training has not been clearly linked to outcomes [61].

## Conclusion

The results and impacts of the current trial on the mindfulness field parallel those in the CBT field following Jacobson’s trial. Before Jacobson et al. [44] dismantled CBT into its separate cognitive and behavioral components, CBT was assumed to improve depression by changing aberrant cognitive schemas. However, Jacobson et al. [44] showed that the effect of the behavioral component alone was no different than that of a full CBT treatment, suggesting that the behavioral component might play a more important role than was previously thought.

Similarly, in the current trial the combination of FA and OM in MBCT did not confer a clear advantage over either component alone. Furthermore, the approach orientation (training attention toward difficulty) that is considered central to the efficacy of MBPs [13] was not superior to training attention away from difficulty. Rather, the approach orientation exemplified by OM was associated with the slowest rate of improvement and lack of efficacy for anxiety. Conversely, the accelerated improvements exhibited by FA on nearly all measures of affective disturbance, along with fewer deteriorations in stress and anxiety, suggest that this component of mindfulness training may deserve more consideration in future implementation of MBPs. While the efficacy of MBCT for the treatment of depression remains intact, this study raises questions about the assumed mechanisms of action and whether they equally apply to conditions other than depression. Together, these findings provide both new perspectives on and opportunities for maximizing the efficacy of MBPs.

## Supporting information

S1 Checklist (DOC)

S1 Table Between-group differences in stress, anxiety and depression at weeks 8 and 20. (DOCX)

S2 Table Within-group differences in stress, anxiety and depression comparing baseline to weeks 8 and 20. (DOCX)

S1 Appendix Reliable change index. (DOCX)

S2 Appendix Trial protocol. (PDF)

S1 Dataset (XLSX)
