# Supplementary material for: The contributions of focused attention and open monitoring in mindfulness-based cognitive therapy for affective disturbances: A 3-armed randomized dismantling trial
Source: PLoS One. 2021 Jan 12;16(1):e0244838. doi: 10.1371/journal.pone.0244838 (PMC7802967; doi:10.1371/journal.pone.0244838)
Supplement: S1 Table — (DOCX) [file pone.0244838.s002.docx]

S2 Table

*Between-group differences in DASS stress, anxiety and depression at weeks 8 and 20*

| Measure | Week | Contrast | *b* | SE | 95% CI | *p*^b^ | *d* |
| --- | --- | --- | --- | --- | --- | --- | --- |
| DASS  Stress^a^ | 8 | FA vs OM | -0.53 | 1.45 | [-3.41, 2.35] | 0.99 | -0.05 |
|  |  | FA vs MBCT | -0.32 | 1.45 | [-3.2, 2.56] | 0.99 | -0.03 |
|  |  | OM vs MBCT | 0.21 | 1.50 | [-2.76, 3.19] | 0.99 | 0.02 |
|  | 20 | FA vs OM | -2.04 | 1.55 | [-5.11, 1.03] | 0.64 | -0.16 |
|  |  | FA vs MBCT | -0.03 | 1.54 | [-3.08, 3.03] | 0.99 | -0.002 |
|  |  | OM vs MBCT | 2.01 | 1.60 | [-1.17, 5.2] | 0.64 | 0.16 |
| DASS Anxiety^a^ | 8 | FA vs OM | -1.31 | 0.68 | [-2.66, 0.04] | 0.38 | -0.24 |
|  |  | FA vs MBCT | -0.40 | 0.67 | [-1.73, 0.93] | 0.99 | -0.07 |
|  |  | OM vs MBCT | 0.92 | 0.70 | [-0.48, 2.31] | 0.64 | 0.17 |
|  | 20 | FA vs OM | -2.25 | 0.78 | [-3.79, -0.71] | 0.06 | -0.36 |
|  |  | FA vs MBCT | 0.25 | 0.76 | [-1.26, 1.76] | 0.99 | 0.04 |
|  |  | OM vs MBCT | 2.50 | 0.80 | [0.90, 4.09] | 0.06 | 0.40 |
| DASS Depression^a^ | 8 | FA vs OM | -0.02 | 1.07 | [-2.15, 2.11] | 0.99 | -0.003 |
|  |  | FA vs MBCT | 0.25 | 1.08 | [-1.89, 2.38] | 0.99 | 0.03 |
|  |  | OM vs MBCT | 0.27 | 1.10 | [-1.91, 2.45] | 0.99 | 0.03 |
|  | 20 | FA vs OM | -0.30 | 1.60 | [-3.48, 2.88] | 0.99 | -0.02 |
|  |  | FA vs MBCT | 2.80 | 1.59 | [-0.36, 5.96] | 0.39 | 0.22 |
|  |  | OM vs MBCT | 3.11 | 1.65 | [-0.18, 6.39] | 0.38 | 0.24 |
| Depression IDS^a^ | 8 | FA vs OM | -0.13 | 1.46 | [-3.03, 2.76] | 0.99 | -0.01 |
|  |  | FA vs MBCT | -0.41 | 1.47 | [-3.33, 2.51] | 0.99 | -0.03 |
|  |  | OM vs MBCT | -0.28 | 1.52 | [-3.31, 2.75] | 0.99 | -0.02 |
|  | 20 | FA vs OM | -0.27 | 1.64 | [-3.52, 2.99] | 0.99 | -0.02 |
|  |  | FA vs MBCT | 0.25 | 1.65 | [-3.03, 3.54] | 0.99 | 0.02 |
|  |  | OM vs MBCT | 0.52 | 1.71 | [-2.87, 3.91] | 0.99 | 0.04 |

*Note.* FA = Focused attention; OM = Open monitoring; MBCT = Mindfulness-based Cognitive Therapy; IDS = Inventory of Depressive Symptomatology; DASS = Depression Anxiety Stress Scales; *b* = estimated mean difference between groups; SE=standard error; CI = confidence interval; *d* = Cohen’s *d*.

^a^Baseline adjusted. ^b^False discovery rate (FDR) adjusted *p* value
