# Supplementary material for: The contributions of focused attention and open monitoring in mindfulness-based cognitive therapy for affective disturbances: A 3-armed randomized dismantling trial
Source: PLoS One. 2021 Jan 12;16(1):e0244838. doi: 10.1371/journal.pone.0244838 (PMC7802967; doi:10.1371/journal.pone.0244838)
Supplement: S2 Table — (DOCX) [file pone.0244838.s003.docx]

S3 Table

*Within-group differences in DASS stress, anxiety and depression comparing baseline to weeks 8 and 20*

| Measure | | *b* | *SE* | *p*^a^ | 95% CI | *d* |
| --- | --- | --- | --- | --- | --- | --- |
| Depression (IDS) | |  |  |  |  |  |
| week 8 | OM | -12.11 | 1.39 | < .001 | [-14.86, -9.36] | 1.65 |
|  | MBCT | -11.86 | 1.44 | < .001 | [-14.71, -9.01] | 1.42 |
|  | FA | -11.81 | 1.33 | < .001 | [-14.44, -9.17] | 1.48 |
| week 20 | OM | -12.07 | 1.45 | < .001 | [-14.94, -9.20] | 1.57 |
|  | MBCT | -12.54 | 1.49 | < .001 | [-15.50, -9.58] | 1.34 |
|  | FA | -11.87 | 1.39 | < .001 | [-14.63, -9.12] | 1.53 |
| Stress (DASS) | |  |  |  |  |  |
| week 8 | OM | -5.92 | 1.34 | < .001 | [-8.59, -3.26] | 0.65 |
|  | MBCT | -5.07 | 1.38 | < .001 | [-7.82, -2.32] | 0.64 |
|  | FA | -5.71 | 1.30 | < .001 | [-8.29, -3.13] | 0.90 |
| week 20 | OM | -4.84 | 1.31 | < .001 | [-7.45, -2.24] | 0.55 |
|  | MBCT | -5.77 | 1.34 | < .001 | [-8.42, -3.12] | 0.74 |
|  | FA | -6.21 | 1.25 | < .001 | [-8.69, -3.74] | 0.92 |
| Anxiety (DASS) | |  |  |  |  |  |
| week 8 | OM | -2.08 | 0.66 | .003 | [-3.39, -0.76] | 0.43 |
|  | MBCT | -1.40 | 0.68 | .050 | [-2.75, -0.04] | 0.42 |
|  | FA | -1.89 | 0.64 | .005 | [-3.17, -0.62] | 0.55 |
| week 20 | OM | -1.12 | 0.65 | .090 | [-2.41, 0.17] | 0.27 |
|  | MBCT | -2.22 | 0.66 | .002 | [-3.53, -0.91] | 0.65 |
|  | FA | -2.00 | 0.61 | .002 | [-3.22, -0.78] | 0.58 |
| Depression (DASS) | |  |  |  |  |  |
| week 8 | OM | -5.94 | 1.09 | < .001 | [-8.11, -3.78] | 1.04 |
|  | MBCT | -5.54 | 1.13 | < .001 | [-7.77, -3.3] | 0.81 |
|  | FA | -4.08 | 1.06 | < .001 | [-6.18, -1.97] | 0.78 |
| week 20 | OM | -4.05 | 1.40 | .006 | [-6.83, -1.26] | 0.56 |
|  | MBCT | -6.13 | 1.43 | < .001 | [-8.97, -3.29] | 0.71 |
|  | FA | -2.26 | 1.34 | .090 | [-4.92, 0.39] | 0.30 |

*Note.* FA = Focused attention; OM = Open monitoring; MBCT = Mindfulness-based Cognitive Therapy; IDS = Inventory of Depressive Symptomatology; DASS = Depression Anxiety Stress Scales; *b* =estimated mean difference compared to baseline; CI = confidence interval; *d* = Within-group Cohen’s *d.*

^a^False discovery rate (FDR) adjusted *p* value
