# Supplementary material for: The contributions of focused attention and open monitoring in mindfulness-based cognitive therapy for affective disturbances: A 3-armed randomized dismantling trial
Source: PLoS One. 2021 Jan 12;16(1):e0244838. doi: 10.1371/journal.pone.0244838 (PMC7802967; doi:10.1371/journal.pone.0244838)
Supplement: S1 Appendix — (DOCX) [file pone.0244838.s004.docx]

**S4 Appendix**

**Reliable Change Index (RCI)**

**Reliable change and clinical significance:**

‘Reliable clinical significance’ of intervention effects was defined by meeting the following two criteria:

1) reliable change scores as indicated by the Reliable Change Index (RCI),

2) improvement in severity level of outcome measures, IDS and DASS (anxiety, depression, stress).

(See Table S4.1 for severity cutoff scores for each instrument)

If criteria 1 and 2 did not agree for a given outcome, then it was not counted as clinically significant improvement or deterioration.

The RCI was calculated based on the formula, **RCI=(X_a_-X_0_)/S_diff_** , where, X_a_= scale score at one of the time point after baseline, X_0_: baseline scale score, S_diff_ =√2*Se^2^, Se = SD*√(1-r), SD is the standard deviation and r is the reliability measure of the measurement scale at baseline (Jacobson, Roberts, Berns, & McGlinchey, 1999; Jacobson & Truax, 1991).

Based on the two above factors, the change scores from baseline to week 8 and 20 of the IDS and week 2, 4, 6, 8 and 20 of DASS anxiety, stress and depression scores in each treatment were classified into 3 categories:

(1) reliable improvement (RCI≤-1.96 and moving from a higher level to a lower level),

(2) no reliable change (-1.96<RCI <1.96) or non clinically significant changes (stay in the same level of severity), or

(3) reliable deterioration (RCI≥-1.96 and moving from a lower level to a higher level)

Table S4.1

*Severity cutoffs for IDS and DASS*

|  | normal | mild | moderate | severe | very severe |
| --- | --- | --- | --- | --- | --- |
| IDS | 0-13 | 14-25 | 26-38 | 39-48 | 49-84 |
| DASS Depression | 0-9 | 10-13 | 14-20 | 21-27 | 28+ |
| DASS Anxiety | 0-7 | 8-9 | 10-14 | 15-19 | 20+ |
| DASS Stress | 0-14 | 15-18 | 19-25 | 26-33 | 34+ |

*Note.* IDS = Inventory of Depressive Symptomatology; DASS = Depression, Anxiety Stress Scales

Table S4.2

*RCI values for IDS at week 8 and 20 for each treatment*

|  | FA (*n* = 36) | | | | MBCT (*n* = 32) | | | | OM (*n* = 36) | | | |
| --- | --- | --- | --- | --- | --- | --- | --- | --- | --- | --- | --- | --- |
|  | Week 8 | | Week 20 | | Week 8 | | Week 20 | | Week 8 | | Week 20 | |
| RCI category | *n* | % | *n* | % | *n* | % | *n* | % | *n* | % | *n* | % |
| Improvement | 27 | 75.0 | 26 | 74.3 | 18 | 60.0 | 19 | 63.3 | 25 | 80.7 | 22 | 71.0 |
| No change | 8 | 22.2 | 9 | 25.7 | 12 | 40.0 | 11 | 36.7 | 5 | 16.1 | 9 | 29.0 |
| Deterioration | 1 | 2.8 | 0 | 0.0 | 0 | 0.0 | 0 | 0.0 | 1 | 3.2 | 0 | 0.0 |

*Note.* RCI categories are determined for each timepoint relative to baseline (week 0). RCI = Reliable Change Index; FA = Focused attention; OM = Open monitoring; MBCT = Mindfulness-based Cognitive Therapy

Table S4.3

*RCI values for Depression, Anxiety, and Stress (DASS)*

| **Stress** | FA (*n* = 36) | | | | | | | | | | MBCT (*n* = 32) | | | | | | | | | | OM (*n* = 36) | | | | | | | | | |
| --- | --- | --- | --- | --- | --- | --- | --- | --- | --- | --- | --- | --- | --- | --- | --- | --- | --- | --- | --- | --- | --- | --- | --- | --- | --- | --- | --- | --- | --- | --- |
|  | Week 2 | | Week 4 | | Week 6 | | Week 8 | | Week 20 | | Week 2 | | Week 4 | | Week 6 | | Week 8 | | Week 20 | | Week 2 | | Week 4 | | Week 6 | | Week 8 | | Week 20 | |
|  | *n* | % | *n* | % | *n* | % | *n* | % | *n* | % | *n* | % | *n* | % | *n* | % | *n* | % | *n* | % | *n* | % | *n* | % | *n* | % | *n* | % | *n* | % |
| Improvement | 5 | 14.3 | 12 | 34.3 | 11 | 31.4 | 11 | 32.4 | 11 | 31.4 | 2 | 6.9 | 7 | 23.3 | 4 | 14.8 | 11 | 36.7 | 10 | 33.3 | 5 | 14.7 | 7 | 21.9 | 10 | 33.3 | 10 | 33.3 | 8 | 27.6 |
| No change | 30 | 85.7 | 23 | 65.7 | 24 | 68.6 | 23 | 67.7 | 24 | 68.6 | 23 | 79.3 | 23 | 76.7 | 20 | 74.1 | 18 | 60.0 | 18 | 60.0 | 26 | 76.5 | 21 | 65.6 | 18 | 60.0 | 18 | 60.0 | 19 | 65.5 |
| Deterioration | 0 | 0.0 | 0 | 0.0 | 0 | 0.0 | 0 | 0.0 | 0 | 0.0 | 4 | 13.8 | 0 | 0.0 | 3 | 11.1 | 1 | 3.3 | 2 | 6.7 | 3 | 8.8 | 4 | 12.5 | 2 | 6.7 | 2 | 6.7 | 2 | 6.9 |

| **Anxiety** | FA (*n* = 36) | | | | | | | | | | MBCT (*n* = 32) | | | | | | | | | | OM (*n* = 36) | | | | | | | | | |
| --- | --- | --- | --- | --- | --- | --- | --- | --- | --- | --- | --- | --- | --- | --- | --- | --- | --- | --- | --- | --- | --- | --- | --- | --- | --- | --- | --- | --- | --- | --- |
|  | Week 2 | | Week 4 | | Week 6 | | Week 8 | | Week 20 | | Week 2 | | Week 4 | | Week 6 | | Week 8 | | Week 20 | | Week 2 | | Week 4 | | Week 6 | | Week 8 | | Week 20 | |
|  | *n* | % | *n* | % | *n* | % | *n* | % | *n* | % | *n* | % | *n* | % | *n* | % | *n* | % | *n* | % | *n* | % | *n* | % | *n* | % | *n* | % | *n* | % |
| Improvement | 6 | 17.1 | 3 | 8.6 | 5 | 14.3 | 5 | 14.7 | 5 | 14.3 | 1 | 3.5 | 2 | 6.7 | 1 | 3.7 | 3 | 10.0 | 3 | 10.0 | 4 | 11.8 | 3 | 9.4 | 2 | 6.7 | 4 | 13.3 | 2 | 6.9 |
| No change | 29 | 82.9 | 32 | 91.4 | 29 | 82.9 | 29 | 85.3 | 30 | 85.7 | 24 | 82.8 | 28 | 93.3 | 22 | 81.5 | 26 | 86.7 | 27 | 90.0 | 27 | 79.4 | 26 | 81.3 | 26 | 86.7 | 26 | 86.7 | 27 | 93.1 |
| Deterioration | 0 | 0.0 | 0 | 0.0 | 1 | 2.9 | 0 | 0.0 | 0 | 0.0 | 4 | 13.8 | 0 | 0.0 | 4 | 14.8 | 1 | 3.3 | 0 | 0.0 | 3 | 8.8 | 3 | 9.4 | 2 | 6.7 | 0 | 0.0 | 0 | 0.0 |

| **Depression** | FA (*n* = 36) | | | | | | | | | | MBCT (*n* = 32) | | | | | | | | | | OM (*n* = 36) | | | | | | | | | |
| --- | --- | --- | --- | --- | --- | --- | --- | --- | --- | --- | --- | --- | --- | --- | --- | --- | --- | --- | --- | --- | --- | --- | --- | --- | --- | --- | --- | --- | --- | --- |
|  | Week 2 | | Week 4 | | Week 6 | | Week 8 | | Week 20 | | Week 2 | | Week 4 | | Week 6 | | Week 8 | | Week 20 | | Week 2 | | Week 4 | | Week 6 | | Week 8 | | Week 20 | |
|  | *n* | % | *n* | % | *n* | % | *n* | % | *n* | % | *n* | % | *n* | % | *n* |  | *n* | % | *n* | % | *n* | % | *n* | % | *n* | % | *n* | % | *n* | % |
| Improvement | 3 | 8.6 | 7 | 20.0 | 6 | 17.1 | 8 | 23.5 | 7 | 20.0 | 4 | 13.8 | 8 | 26.7 | 8 | 29.6 | 10 | 33.3 | 11 | 36.7 | 6 | 17.6 | 8 | 25.0 | 9 | 30.0 | 8 | 26.7 | 7 | 24.1 |
| No change | 32 | 91.4 | 28 | 80.0 | 28 | 80.0 | 25 | 73.5 | 24 | 68.6 | 23 | 79.3 | 21 | 70.0 | 15 | 55.6 | 20 | 66.7 | 18 | 60.0 | 25 | 73.5 | 20 | 62.5 | 21 | 70.0 | 22 | 73.3 | 20 | 69.0 |
| Deterioration | 0 | 0.0 | 0 | 0.0 | 1 | 2.9 | 1 | 2.9 | 4 | 11.4 | 2 | 6.9 | 1 | 3.3 | 4 | 14.8 | 0 | 0.0 | 1 | 3.3 | 3 | 8.8 | 4 | 12.5 | 0 | 0.0 | 0 | 0.0 | 2 | 6.9 |

*Note.* RCI categories are determined for each timepoint relative to baseline (week 0). RCI = Reliable Change Index; FA = Focused attention; OM = Open monitoring; MBCT = Mindfulness-based Cognitive Therapy

**Figures S4.1 - S4.4: RCI Scatterplots**

**Figure S4.1. RCI for IDS at weeks 8 and 20**

**Figure S4.2. RCI Plots for DASS Stress at weeks 2, 4, 6, 8 and 20**

**Figure S4.3. RCI Plots for DASS Anxiety at weeks 2, 4, 6, 8 and 20**

**Figure S4.4. RCI Plots for DASS Depression at weeks 2, 4, 6, 8 and 20**

Plus symbols (+) signify reliable improvements. X symbols (x) signify reliable deteriorations. Circle symbols (○) signify “no reliable change”. The area between two dash lines represent i.e, -1.96 ≤ RCI ≤ 1.96 (no reliable change). Points that were outside this area but in the same level of severity at baseline were classified also classified as “no reliable change”, and also represented with a circle (○).

**Figure S4.1. RCI plots for IDS at Week 8 and Week 20 [top=OM; middle=MBCT; bottom=FA]**

|  |  |
| --- | --- |
|  |  |
|  |  |

**Figure S4.2. RCI plots for DASS Stress [left=OM; middle=MBCT; right=FA]**

|  |  |  |
| --- | --- | --- |
|  |  |  |
|  |  |  |
|  |  |  |
|  |  |  |

**Figure S4.3. RCI plots for DASS Anxiety [left=OM; middle=MBCT; right=FA]**

|  |  |  |
| --- | --- | --- |
|  |  |  |
|  |  |  |
|  |  |  |
|  |  |  |

**Figure S4.4. RCI plots for DASS Depression [left=OM; middle=MBCT; right=FA]**

|  |  |  |
| --- | --- | --- |
|  |  |  |
|  |  |  |
|  |  |  |
|  |  |  |
