# Supplementary material for: The contributions of focused attention and open monitoring in mindfulness-based cognitive therapy for affective disturbances: A 3-armed randomized dismantling trial
Source: PLoS One. 2021 Jan 12;16(1):e0244838. doi: 10.1371/journal.pone.0244838 (PMC7802967; doi:10.1371/journal.pone.0244838)
Supplement: S2 Appendix — (PDF) [file pone.0244838.s005.pdf]

TITLE

Dismantling Mindfulness: Contributions of Attention vs. Acceptance

NCCAM Protocol Number: 1 K23 AT006328-01A1

Sponsored by:

National Center for Complementary and Alternative Medicine

NCCAM Funding Mechanism:

MENTORED PATIENT-ORIENTED RESEARCH CAREER DEVELOPMENT AWARD
K23 AT006328-01A1

Principal Investigator:

Willoughby Britton, Ph.D.
Department: Psychiatry and Human Behavior
Brown University Medical School
Providence RI 02906

Draft or Version Number: 3.3

Day Month Year

November 5 2012

This template is adapted from the ICH guidance document E6 (Good Clinical Practices), Section 6.

Confidentiality Statement

This document is confidential and is to be distributed for review only to investigators, potential investigators, consultants, study staff, and applicable independent ethics committees or institutional review boards.

Table of Contents - *continued*

LIST OF ABBREVIATIONS V
1. PERSONNEL + SITES VII
PROTOCOL SUMMARY VIII
2. INTRODUCTION 10
2.1 PATIENT ORIENTED MENTORED CAREER DEVELOPMENT AWARD 10
2.2 SPECIFIC AIMS 10
3. BACKGROUND AND SCIENTIFIC RATIONALE 11
3.1 BACKGROUND INFORMATION 11
3.2 POTENTIAL RISKS AND BENEFITS 15
3.2.1 Potential Risks 15
3.2.2 Potential Benefits 15
4. STUDY OBJECTIVES 16
4.1 PRIMARY OBJECTIVE 16
4.2 SECONDARY OBJECTIVES 16
4.3 EXPLORATORY OBJECTIVES 16
5. STUDY DESIGN 16
5.1 FLOW CHART FOR STUDY DESIGN 17
5.2 DESCRIPTION OF THE STUDY DESIGN 17
5.3 STUDY ENDPOINTS 19
5.3.1 Primary clinical Endpoint 19
5.3.2 Secondary Endpoints 19
5.3.3 Exploratory endpoints 20
6. STUDY POPULATION 20
6.1 OVERVIEW 20
6.2 INCLUSION CRITERIA: 20
6.3 EXCLUSION CRITERIA: 20
6.4 INCLUSION OF WOMEN AND MINORITIES 21
6.5 INCLUSION OF CHILDREN 21
6.6 TARGET/PLANNED ENROLLMENT 22
6.7 CO-ENROLLMENT GUIDELINES 22
6.8 STRATEGIES FOR RECRUITMENT AND RETENTION 23
7. METHODS FOR MINIMIZING BIAS 23
7.1 TREATMENT RANDOMIZATION 23
7.1.1 Background Rationale 23
7.1.2 Clinical and Treatment Considerations 23
7.1.3 Feasibility and practical considerations 23
7.1.4 Randomization Procedures 24
7.1.4.1 Introduction 24
7.1.4.2 Timing: 24
7.1.4.3 Group Randomization 24
7.2 BLINDING 25
7.3 RELIABILITY 25
8. STUDY INTERVENTIONS 25
8.1 TREATMENT MATCHING 25
8.2 STUDY INTERVENTION DESCRIPTIONS 26

Table of Contents - *continued*

8.2.1 Intervention #1: MBCT ... 26
8.2.2 Intervention #2: Focused Awareness ... 26
8.2.3 Intervention #3: Open Monitoring ... 26
8.3 THERAPIST QUALIFICATIONS AND TRAINING ... 26
8.3.1 Qualifications: ... 26
8.3.2 Training ... 27
8.4 THERAPIST COMPETENCY, TREATMENT FIDELITY AND ADHERENCE ... 27
8.4.1 Adherence rating by trained judges: ... 27
8.4.2 Ratings by Participants: ... 27
8.5 PARTICIPANT COMPLIANCE WITH INTERVENTION(S) ... 27
9. STUDY SCHEDULE ... 28
9.1 SCREENING ... 28
9.1.1 Phone screen ... 28
9.1.2 In person interview ... 28
9.1.2.1 Concomitant Medications and Procedures ... 29
9.1.2.2 Provisional inclusions: ... 29
9.1.3 Exclusion Procedures: ... 29
9.2 INFORMED CONSENT ... 29
9.3 BASELINE ASSESSMENT ... 30
9.4 ENROLLMENT ... 30
9.5 TREATMENT RANDOMIZATION ... 30
9.6 TREATMENT ... 30
9.7 POST TREATMENT ASSESSMENT ... 30
9.8 3 MONTH FOLLOW-UP ASSESSMENT ... 30
9.9 ADVERSE EFFECTS ... 31
9.9.1 Stopping Rules for an Individual Participant ... 31
10. ASSESSMENT OF SAFETY ... 31
10.1 ETHICS/PROTECTION OF HUMAN SUBJECTS ... 31
10.1.1 Institutional Review Board/Ethics Committee ... 31
10.1.2 Informed Consent Process: ... 31
10.1.3 Adherence ... 32
10.1.4 Attached IRB Documents: ... 32
10.2 ADVERSE EVENTS ... 32
10.2.1 Definition of an Adverse Event (AE) ... 32
10.2.2 Definition of a Serious Adverse Event (SAE) ... 32
10.2.2.1 AE/SAE Severity ... 33
10.2.2.2 Relationship of AEs/SAEs to the Study Intervention ... 33
10.2.2.3 Expected Risks -NCCAM statement ... 34
10.2.2.4 Impact statement ... 35
10.2.2.5 Measures that will be taken to minimize study risk: ... 35
10.3 DATA COLLECTION PROCEDURES FOR ADVERSE EVENTS ... 36
10.3.1 Scheduled collection ... 36
10.3.2 Unscheduled collection ... 37
10.4 RECORDING/DOCUMENTATION ... 37
10.4.1 AE Reporting and Follow-Up ... 37
10.4.2 Reporting Procedures ... 38

Table of Contents - *continued*

10.4.2.1 SAEs ... 38
10.4.2.2 Changes in Study Status ... 38
10.5 STOPPING RULES FOR THE PROTOCOL ... 38
10.5.1 Stopping Rules for an Individual Participant ... 39
10.6 DSMB INDEPENDENT MONITORING COMMITTEE ... 39
10.6.1 Members of Monitoring Committee ... 39
10.6.2 Safety Review Plan ... 40
10.6.3 Subject Accrual and Compliance ... 40
10.6.3.1 Measurement and Reporting of Subject Accrual, Compliance with Inclusion/Exclusion Criteria: ... 40
10.6.3.2 Measurement and Reporting of Participant Adherence to Treatment Protocol ... 40
10.6.4 Frequency of Data Review for this Study ... 41
10.6.4.1 Study Report Outline for the Independent Monitors(s) (Interim or Annual Reports) ... 41
11. STATISTICAL CONSIDERATIONS ... 41
11.1 STUDY OVERVIEW: ... 42
11.2 OBJECTIVES AND HYPOTHESES: ... 42
11.3 ANALYSIS PLAN: ... 42
11.3.1 Preliminary Analyses: ... 42
11.3.2 Primary Analyses: ... 42
11.3.3 Secondary Analysis: ... 43
11.3.3.1 To compare the effects of FA, OM and MBCT on attention, emotion regulation and wakefulness: ... 43
11.3.3.2 To determine which neurobehavioral process drives the clinical benefits of mindfulness: ... 44
11.4 SAMPLE SIZE CONSIDERATIONS ... 44
11.4.1 Objectives ... 44
11.4.2 Feasibility limitations – maximum capacity ... 44
11.4.3 Relevant pilot data: ... 44
11.4.4 Basic Calculations ... 44
11.4.5 Additional Sample size considerations +modifications ... 45
11.4.5.1 Attrition ... 45
11.4.5.2 Sample size adjustment due to group randomization ... 45
11.4.6 Multiple comparisons ... 45
11.4.7 Minimum Sample Size ... 45
11.5 PLANNED INTERIM ANALYSES (IF APPLICABLE) ... 46
11.6 MISSING DATA ... 46
12. DATA HANDLING AND RECORD KEEPING ... 46
12.1 TYPES OF DATA: ... 46
12.2 DATA CONFIDENTIALITY: ... 46
12.3 DATA STORAGE: ... 47
12.4 DATA VERIFICATION AND REVIEW ... 47
12.5 STUDY RECORDS RETENTION ... 47

LIST OF ABBREVIATIONS	
**AE**	Adverse Event
**BAM**	Beliefs about Meditation
**BFI**	Big Five Personality Inventory
**BTQ**	Buddhist Temperament Questionnaire
**CTQ**	Child Trauma Questionnaire
**DASS**	The Depression, Anxiety Stress Scale
**DERS**	Difficulties in Emotion Regulation Scale
**DSMB**	Data Safety Monitoring Board
**EEG**	Electroencephalography
**EMG**	Electromyography
**ERRT**	Emotion Reactivity and Regulation Task
**ES**	Empathy Scale
**FA**	Focused Awareness
**FFMQ**	Five Facet Mindfulness Questionnaire
**IDS**	Inventory of Depressive Symptomatology-
**IMC**	Independent Monitoring Committee
**KSS**	Karolinska Sleepiness Scale
**MAAS**	Mindful Attention Awareness Scale
**MBCT**	Mindfulness-Based Cognitive Therapy
**MBSR**	Mindfulness-Based Stress Reduction
**MEDEX-I**	Meditation Experiences Interview
**MSAS**	Mindfulness Skill Acquisition Scale
**MSPSS**	Multidimensional Scale of Perceived Social Support
**NCCAM**	National Center for Complementary and Alternative Medicine
**NIH**	National Institutes of Health
**OM**	Open Monitoring
**PANAS**	Positive and Negative Affect Schedule
**PSG**	polysomnography
**RA**	Research Assistant
**SAE**	Serious Adverse Event
**SART**	Sustained Attention to Response Task
**SCID**	Structured Clinical Interview for DSM-IV
**SCO**	Self-Compassion Scale
**SLT**	Sleep Latency test
**SPS**	Spiritual Perspectives Scale
**SRT**	Self-referencing Task
**TFI**	Therapeutic Factors Inventory

**WAI**	The Working Alliance Inventory
**WBS**	Wellbeing Scale

1. PERSONNEL + SITES

Principal Investigator:
Willoughby Britton Ph.D. Brown University

Mentors:
Ivan Miller Ph.D Brown University
Mary Carskadon Ph.D Brown University

Consultants:
Ronald Seifer Ph.D Brown University
Harold Roth Ph.D Brown University
Laurence Hirshberg Ph.D NeuroDevelopment Center
Zindel Segal Centre for Addiction and Mental Health
John Allen University of Arizona
Amishi Jha Ph.D University of Pennsylvania

SITES:

•Psychosocial Research Unit, Department of Psychiatry and Human Behavior, Butler Hospital, 345 *Blackstone* Blvd. Providence, RI (Director: Ivan Miller, PhD)

• The Neurodevelopment Center, 260 West Exchange Street, Suite 210, Providence, RI Director (Director: Lawrence Hirschberg, PhD)

Protocol Summary

**Full Title**	Dismantling Mindfulness: Contributions of Attention vs. Acceptance
**Short Title**	Dismantling Mindfulness:
**Conducted By**	Department of Psychiatry and Human Behavior, Brown University
**Principal Investigator**	Willoughby Britton
**Sample Size**	*105*
**Study Population**	Individuals with mild-severe affective disturbances (depression)
**Accrual Period**	3 years
**Study Design**	Three-armed clinical trial. Groups of participants will be randomly allocated into either a(n) i.) focused awareness (FA), ii.) open monitoring (FA), or iii.) traditional MBCT arm.
**Study Duration**	Career development award: 5 years, 07/01/2011- 06/30/2016<br>Data collection: 3 years<br>6 months per participant: 8 week intervention and 3 month follow-up.
**Intervention Description**	One of three types of meditation: 1) focused awareness 2) open monitoring , or 3) Mindfulness-Based Cognitive Therapy (both practices combined)<br><br>Intervention is group format, 2.5 hours once a week for 8 weeks.
**Primary Objective of this Award**	The purpose of the research described in this Mentored Patient-Oriented Research Career Development Award (K23) is to facilitate my becoming an independent investigator.<br><br>Study the Effects of Different Types of Meditation on Depression and Negative Affect
**Primary Research Objective**	• To create two separate treatment modules with specific criteria for the two core components and practices of attention training/focused awareness (FA) and acceptance/open monitoring (OM); establish feasibility and acceptability<br><br>• To establish the effect sizes of FA and OM vs MBCT in a controlled trial in

	individuals with mild-severe depression and persistent negative affect.
**Secondary Research Objectives**	• To assess and compare the effects of FA, OM, and MBCT on subjective and objective measures of 3 domains of PFC functioning: 1) attention, 2) emotional reactivity/regulation and 3) wakefulness.<br><br>• To determine which neurobehavioral processes (attention, emotion regulation, wakefulness) drive the clinical benefits of mindfulness training, independent of treatment allocation or practice type.
**Endpoints**	1)Depression/Negative Affect:<br>2)Attention:<br>3) Emotional Reactivity/Regulation:<br>4) Wakefulness/Sleepiness**:**

2. INTRODUCTION

2.1 Patient Oriented Mentored Career Development Award

The research project described in this protocol is part of Career Development Award. The primary aim of the research is to provide the PI with training in conducting clinical intervention research, and to provide pilot data for a larger trial (R01). Thus the primary goal of this award (training) is a central part of the design considerations for the research study. It should also be noted that $25,000 per year is available for research, which covers the cost of one paid research assistant. Thus, feasibility should also take into account the limited budget.

2.2 Specific Aims

The primary aim of this K23 proposal is to provide hands-on training to facilitate my becoming an independent investigator in patient-oriented meditation research with an emphasis in mechanisms of action.

Meditation, particularly mindfulness meditation, is one of the most popular Complementary and Alternative Medicine (CAM) therapies for alleviating mild-severe emotional stress, depression and anxiety and promoting wellbeing (NCCAM, 2009a; Schroevers & Brandsma, 2009). However, despite the widespread application and proliferation, numerous methodological limitations in available research preclude definitive claims about clinical effectiveness or mechanism of action (Davidson, in press; Ludwig & Kabat-Zinn, 2008; Ospina et al., 2007). At present, the largest barrier to meditation research in general, and mindfulness research in particular, is the failure to delineate meditation based-treatment packages into their most basic components and practices (Davidson, 2010)(Davidson, in press; Ospina et al., 2007).

Mindfulness has been defined as being comprised of two components: 1) attention training component and 2) an affective orientation of acceptance/non-reactivity (Bishop et al., 2004; Rapgay & Bystrisky, 2009). These two components correspond to two separate meditation practices, focused awareness (FA) and open monitoring (OM), respectively (Davidson, in press; Lutz et al., 2008; Rapgay & Bystrisky, 2009). FA and OM practices are thought to have different neural underpinnings and different cognitive affective and behavioral consequences (Lutz et al., 2008; Rapgay & Bystrisky, 2009). However, there is much debate in both traditional Buddhist and western science about the relative importance of each component for clinical benefit for the alleviation of affective disturbance (Davidson, in press; Rapgay & Bystrisky, 2009; Shankman, 2008). For example, the role of attention training (FA), which is foundational to nearly all forms and traditions of meditation practice, is increasingly diminished in favor of acceptance in the new wave of mindfulness therapies (Rapgay & Bystrisky, 2009). Since the respective contributions of each component have never been assessed, the recent trend of decreasing the proportion of attention training in mindfulness-based therapies may be eliminating an indispensable ingredient that is key to treatment success. Alternatively, it is possible that programs that demand hours of FA training are misinformed and could allocate treatment time to more efficacious techniques or approaches.

Mindfulness-Based Stress Reduction (MBSR) and its close cousin Mindfulness-Based Cognitive Therapy (MBCT) dedicate approximately half of treatment time to attention training/FA and the other half to acceptance/OM (Santorelli & Kabat-Zinn, 2003; Segal, Williams et al., 2002). However, because these two components and practices are delivered in an

integrated format it is difficult to assess their individual contributions to treatment success or their different mechanisms of action. Therefore, the primary goal of the proposed research is to dismantle MBCT into the separate core components/practices of the attention/FA training and acceptance/OM training and to assess each component and practice independently in a controlled trial in 105 individuals with mild-severe affective disturbances.

It has been proposed that the therapeutic effects of mindfulness meditation are mediated by strengthening prefrontal attention and emotion regulation systems (Chambers et al., 2009; Creswell et al., 2007; Davidson & Lutz, 2008; Hofmann & Asmundson, 2008; Teasdale et al., 1995; Way et al., in press). However, both attention and emotion regulation depend on the more basic prefrontal cortex function of wakefulness, which is currently under-researched in the meditation field. In order to *thoroughly* explore practice-specific mechanisms of action, I aim to investigate the effects of the two components of mindfulness training (FA and OM) on *all three* indices of PFC functioning: 1) attention, 2) emotional regulation *and* 3) wakefulness.

The Specific Aims of the project are:
•**Aim 1:** Provide training in meditation-based clinical trials for affective disturbances, psychophysiology, biostatistics research methods, and ethical conduct in research.
•**Aim 2:** To create two separate treatment modules with specific criteria for the two core components and practices of attention training/focused awareness (FA) and acceptance/open monitoring (OM)
•**Aim 3:** To establish the effect sizes of FA and OM vs the standard MBCT package in a controlled trial in individuals with mild-severe depression and persistent negative affect.
•**Aim 4:** To assess and compare the effects of FA, OM and MBCT on subjective and objective measures of 3 domains of PFC functioning: 1) attention, 2) emotional reactivity/regulation and 3) wakefulness.
•**Aim 5:** To determine which neurobehavioral processes (attention, emotion regulation, wakefulness) drive the clinical benefits of mindfulness training, independent of treatment allocation or practice type.

The findings from this K23 proposal will provide pilot data for a future R01 application for a definitive test of the practice-specific mechanisms of action and contributions to clinical l benefit.

3. BACKGROUND AND SCIENTIFIC RATIONALE

3.1 Background Information

***NCCAM's overarching research priority is** to investigate "CAM interventions used frequently by the American public, and on the conditions for which they are most frequently used" (NCCAM, 2009b)*

Meditation is one the most popular and fastest growing CAM therapies, attracting over a million new meditators per year in the United States alone (Barnes et al., 2008). The main reason Americans use meditation is for the alleviation of emotional distress, including mild-severe depression, anxiety and stress, and to promote wellbeing (NCCAM, 2009a). Meditation-related Medline articles have tripled in the last 3 years and NIH-funded clinical trials of meditation have increased more than 20-fold since 2000.

Meditation's popularity is outpacing empirical support. A 2007 government report on "Meditation Practices for Health: State of the Research" (Ospina et al., 2007) concluded that "as a whole, firm conclusions on the effects of meditation practices in healthcare cannot be drawn based on the available evidence" (pg. 210). The lack of evidence is not due to a paucity of studies (>813 studies reviewed) or a lack of randomized controlled trials (286 RCTs included). **The central obstacle in meditation research is the nearly complete absence of delineation of the different types of meditation practices**, both in terms of their operational descriptions and separate (or additive) effects on outcomes. The report on meditation research concludes, "the field of research on meditation techniques and their therapeutic applications has been clouded by confusion over what constitutes meditation…. Further research needs to be directed toward distinguishing the effects and characteristics of the many different techniques falling under the rubric "meditation"(pg 209) and "systematically comparing the effects of different meditation practices that research shows have promise "(pg. 208). In 2008, NCCAM requested research that "develops precise criteria for specific meditation practices(NCCAM, 2008).
Mindfulness is the fasting growing form of meditation. More than any other form of meditation, mindfulness meditation has shown an unprecedented expansion throughout mainstream psychiatry and has been touted as the "Third Wave of Behavior Therapies"(Rapgay & Bystrisky, 2009).
Mindfulness is comprised of two distinct components and practices that are rarely separated: attention training and emotional non-reactivity or acceptance (Bishop et al., 2004; Rapgay & Bystrisky, 2009). Attention regulation and the ability to sustain attention is thought to be cultivated through the practice of focused awareness (FA) while a non-judgmental, accepting attitude is thought to be cultivated through the practice of open monitoring (OM)(Lutz et al., 2008; Rapgay & Bystrisky, 2009). These two components and practices are delivered in an integrated way in popular therapies like Mindfulness-Based Stress Reduction (MBSR) and Mindfulness-Based Cognitive Therapy (MBCT). However, FA and OM are thought to have different neural underpinnings and different cognitive affective and behavioral consequences (Lutz et al., 2008; Rapgay & Bystrisky, 2009). While both of these components and practices are the foundation of mindfulness-based therapies, it is unknown what contribution each component/practice makes to therapeutic outcome or by what cognitive or biological mechanism. By delineating mindfulness into its separate components, this project will address the largest confound and obstacle in meditation research. By providing knowledge about the relative benefits of different meditation practices, this project will be able to separate the active ingredient(s) from non-beneficial components and practices.

NCCAM Priority: Clarify the neurobiological mechanisms that underlie meditation's impact on mental health and wellbeing (NCCAM, 2008). Knowing that a specific meditation practice is effective at improving affective disturbance is not enough, we also need to know *how* and *why* it is effective.
Meditation is hypothesized to exert its therapeutic effects on affective disturbances by strengthening networks of the prefrontal cortex (PFC) (Chambers et al., 2009; Creswell et al., 2007; Davidson & Lutz, 2008; Hofmann & Asmundson, 2008; Teasdale et al., 1995; Way et al., in press) which regulates emotional reactivity, attention, and wakefulness. This hypothesis is based on the following:
a) Meditation restores PFC deficits and associated affective disturbances: Emotional disturbances characterized by high negative affect are associated with PFC hypoactivity (Clark et

al., 2009; Couyoumdjian et al., 2009), and restoration of PFC functioning parallels response to treatment in both pharmacological and behavioral interventions,(Davidson et al., 2003; Hugdahl et al., 2007; Liotti & Mayberg, 2001; Liotti et al., 2002). Meditation practice, widely defined, is associated with increased activity in the PFC, (Baerentsen, 2001; Brefczynski-Lewis et al., 2007; Creswell et al., 2007; Farb et al., 2007; Herzog et al., 1990; Holzel et al., 2007; Jevning et al., 1996; Khushu et al., 2000; Lazar et al., 2000; Newberg et al., 2001; Ritskes R et al., 2003), larger frontal gray matter volumes (Holzel et al., 2008; Lazar et al., 2005; Luders et al., 2009; Pagnoni & Cekic, 2007) and decreased levels of negative affect, anxiety and depression (Allen et al., 2006; Grossman et al., 2004; Jain et al., 2007; Kabat-Zinn, Massion, Kristeller, Peterson, Fletcher, Pbert, Linderking et al., 1992; Kenny & Williams, 2007; Kuyken et al., 2008; Shapiro et al., 1998; Speca et al., 2000; Witek-Janusek et al., 2008).
b) Meditation improves PFC-related emotional regulation: The PFC exerts inhibitory control on limbic structures such as the amygdala (Davidson et al., 2000; Mayberg et al., 1999; Ochsner et al., 2002; Ochsner & Gross, 2005; Ochsner et al., 2004; Urry et al., 2006). Lack of such inhibitory control results in a hyperactive amygdala (Siegle et al., 2002; Siegle et al., 2007) and an associated increase in emotional reactivity, sympathetic hyperarousal and affective disturbance (Baxter et al., 1989; Bench et al., 1993; Blumberg et al., 2004; Clark et al., 2002; Davidson, 2000; Mayberg et al., 1999; Meyer et al., 2004; Siegle & Hasselmo, 2002; Siegle et al., 2002). Meditation training is associated with greater PFC inhibition of the amygdala (Brefczynski-Lewis et al., 2007; Creswell et al., 2007; Farb et al., 2007; Holzel et al., 2010; Way et al., in press), less emotional reactivity(Arch & Craske, 2006, 2010; Brewer et al., 2009; Broderick, 2005; Campbell-Sills et al., 2006; Creswell et al., 2007; Erisman & Roemer, 2010; Goldin & Gross, 2010; Kuehner et al., 2009; McKee et al., 2007; Ortner et al., 2007; Pace et al., 2009; Proulx, 2008; Raes et al., 2009; Tang et al., 2007; Vujanovic et al., 2007; Weinstein et al., 2009), and decreased sympathetic hyperarousal (Britton, Bootzin, Haynes et al., 2010; Carlson et al., 2007; Maclean et al., 1994; Sudsuang et al., 1991).
c) Meditation improves PFC-related attention capabilities: The PFC is involved in different forms of attention including orienting, executive function and alerting (Fan et al., 2002; Fan et al., 2003). Insufficient PFC activation is associated with deficits in attention, such as "difficulty concentrating and making decisions", which are hallmark symptoms of depression. Meditation training is associated with better performance on a wide range of prefrontally-mediated attention tasks (Brefczynski-Lewis et al., 2007; Bushell, 2009; Chambers et al., 2008; Chan & Woollacott, 2007; Jha et al., 2007; Lutz et al., 2009; Pagnoni & Cekic, 2007; Slagter et al., 2007; Srinivasan & Baijal, 2007; Tang et al., 2007; Valentine & Sweet, 1999; Wenk-Sormaz, 2005).
d) Meditation is associated with increased wakefulness: High levels of blood flow and cerebral metabolic rate in the PFC correspond to alert wakefulness (Buchsbaum et al., 1989; Maquet et al., 1990; Sawaya & Ingvar, 1989), while a hypoactive PFC is associated with fatigue, tiredness and increased sleep propensity (Braun et al., 1997; Buchsbaum et al., 1989; Maquet et al., 1996). Six cross-sectional studies have found EEG-based evidence for increased wakefulness and decreased sleep propensity in meditators (Banquet & Sailhan, 1974; Cahn et al., in press; Cahn & Polich, 2009; Elson et al., 1977; Kaul et al., 2010; Mason et al., 1997) but longitudinal evidence with MBSR interventions is largely based on subjective reports (Carlson & Garland, 2005; Cohen et al., 2004; Singh et al., 1998; Surawy et al., 2005; Tang et al., 2007). In the first objective EEG-based longitudinal RCT of meditation and sleep propensity, I found increased wakefulness and decreased sleep propensity following MBCT that was correlated with improved

depression and meditation practice amount (Britton, Haynes et al., 2010) (see Preliminary Studies section)**.**

Together, these studies support the idea that strengthening PFC functioning plays a key role in meditation's beneficial effects. It is unknown which PFC process (attention, emotion regulation, wakefulness) are the most important. This study aims to investigate which of these *processes* (independent of practice type) drives clinical benefit in affective disturbances.

<u>Current research has obscured the relationship between practice type and mechanism.</u> All of the prior studies listed above have either used cross-sectional studies of experienced practitioners or heterogeneous treatment packages that obscure practice specific effects. It is assumed that FA training improves attention and OM improves emotional reactivity (Lutz et al., 2008), but because these practices have never been separated in research, these assumptions about practice-specific effects are untested.

To test these assumptions about practice specific mechanisms of action, this study will create separate practice criteria for focused awareness and open-monitoring/acceptance practices, compare their clinical efficacy for affective disturbance and investigate their *separate* effects on attention, wakefulness, and emotional reactivity/regulation. By knowing the specific processes that are affected by each practice, clinicians may be able to tailor treatments based on specific needs (attention deficits, emotion regulation problems etc.).

B. INNOVATION

B1. This study is innovative because it addresses multiple methodological limitations of prior studies.

In addition to the central flaws of inadequate practice delineation and understanding of mechanism, mindfulness treatment research has been plagued by a number of other methodological limitations including lack of 1) active control groups 2) objective or physiological measures 3) protocol adherence and participant skill acquisition (treatment fidelity) and lack of generalizability due to condition-specific adaptations (Ludwig & Kabat-Zinn, 2008).

Therefore this study aims to address these barriers by 1) delineating mindfulness into its two core components 2) comparing these components in a controlled trial against the standard (active) treatment 3) using both subjective and objective/physiological measures, 4) carefully operationalizing and monitoring precise treatment criteria adherence and skill acquisition and 5) choosing a sample population that represents the unifying feature of all mindfulness interventions: mild-severe affective disturbance.

***B2: This study is innovative because it is the first study to dismantle mindfulness*.** There will be an 8-week intervention for each component/practice: 1) attention/focused awareness training (FA) and 2) acceptance/open monitoring training (OM), following Jacobson et al.'s classic dismantling paradigm (Jacobson et al., 1996). The study will investigate the effects of individual components/practices on clinical efficacy and mechanism of action in comparison to each other and the standard MBCT program, which includes both components.

B3: This study is innovative because it is the first study to examine the practice-specific effects on the foundational PFC domain of wakefulness, on which both attention and emotion regulation depend. A number of studies have found that meditation is associated with improved PFC functioning, including attentional capabilities, emotion regulation and increased wakefulness (see above). To date, research has focused on attention and emotion regulation as the central mechanisms of meditation, with little regard for the related PFC domain of

wakefulness or sleep propensity. However, attention and emotion regulation are dependent on adequate wakefulness and compromised when wakefulness is low or sleep propensity is high (Banks & Dinges, 2007; Chuah et al., 2006; Couyoumdjian et al., 2009; Dinges et al., 1997; Drummond et al., 1999; Franzen et al., 2008; Goel et al., 2009; Harrison & Horne, 1998; Harrison et al., 2000; Ingre et al., 2006; Lim & Dinges, 2008; Lim et al., 2009; Nilsson et al., 2005; Thase et al., 1997; Van Dongen et al., 2003; Walker, 2009; West et al., 2009; Yoo, Gujar et al., 2007; Yoo, Hu et al., 2007; Zohar et al., 2005). Therefore knowledge of practice-specific effects on wakefulness will provide foundational information for understanding mechanisms of action. In order to provide the most comprehensive understanding of mechanism of action, this study will investigate all 3 domains of PFC functioning: Attention, emotion reactivity/regulation, *and* wakefulness.

B4: If successful, this trial will provide: (a) The first, separate manualized treatment modules for the two core components of mindfulness with precise criteria for FA and OM meditation practices, (b) information about the clinical efficacy of each component/practice alone and in contrast to the standard combined treatment package and (c) information about the specific mechanisms of action for each component/practice. This information will identify *which* practices are most beneficial and *why* they are effective.

3.2 Potential Risks and Benefits

3.2.1 Potential Risks

1) Potential coercion. It is possible that patients may feel coerced into participating.
2) Confidentiality and loss of privacy. We will be collecting considerable information about the subject which may create some distress and could cause social and psychological risk if released inappropriately.
3) Discomfort during meditation: Meditation-based interventions may results in discomfort with attention to unpleasant thoughts, feelings or body sensations. Some individuals may experience an initial increase in undesirable feelings with increased attention to them.
4) Discomfort during assessments: Some participants may feel uncomfortable answering personal questions about their psychological health. The physiological recording electrodes may cause mild skin irritation. Some of the stimuli may be unpleasant or graphic.
5) Risk of adverse events during the study. It is possible that some patients will have an adverse event during the study, including failure to respond to treatment, worsening of depressive symptoms, suicidal ideation or behavior, or other adverse events.

3.2.2 Potential Benefits

A major goal of this study is to assess the differential impact of two commonly used forms of meditation practice. Thus, this project will provide much better information about active ingredients in a popular intervention. Furthermore, these studies may provide information to clinicians about how two common forms of meditation may work differently and may be better

suited for some patients than others. This will allow clinicians to administer the best treatment to respond to patients needs and promote wellbeing.

4. STUDY OBJECTIVES

4.1 Primary Objective

•**<u>Aim 1:</u>** Provide training in meditation-based clinical trials for affective disturbances, psychophysiology, biostatistics research methods, and ethical conduct in research.

•**<u>Aim 2</u>:** To create two separate treatment modules with specific criteria for the two core components and practices of attention training/focused awareness (FA) and open monitoring (OM)

•**<u>Aim 3</u>:** To establish the effect of FA and OM vs MBCT on depression and negative affect

	**Objective**	**Self-report**
Depression/negative affect	IDS interview	DASS
Positive affect/wellbeing		PANAS, WBS

4.2 Secondary Objectives

•**<u>Aim 4:</u>** To assess and compare the effects of FA, OM and MBCT on subjective and objective measures of 3 domains of PFC functioning:

**PFC Domain**	**Objective**	**Self-report**
attention	SART	FFMQ, MAAS
emotional reactivity/regulation	ERRT	DERS, SCO
wakefulness	5-9 HZ EEG power<br>sleep latency	KSS

•**<u>Aim 5</u>:** To determine which neurobehavioral processes (attention, emotion regulation, wakefulness) drive the clinical benefits of mindfulness training, independent of treatment allocation or practice type.

4.3 Exploratory Objectives

To assess factors other than type of mental training that may impact outcome, including therapist-level (competence) and participant level factors (compliance).

5. STUDY DESIGN

5.1 **Flow Chart for study Design**

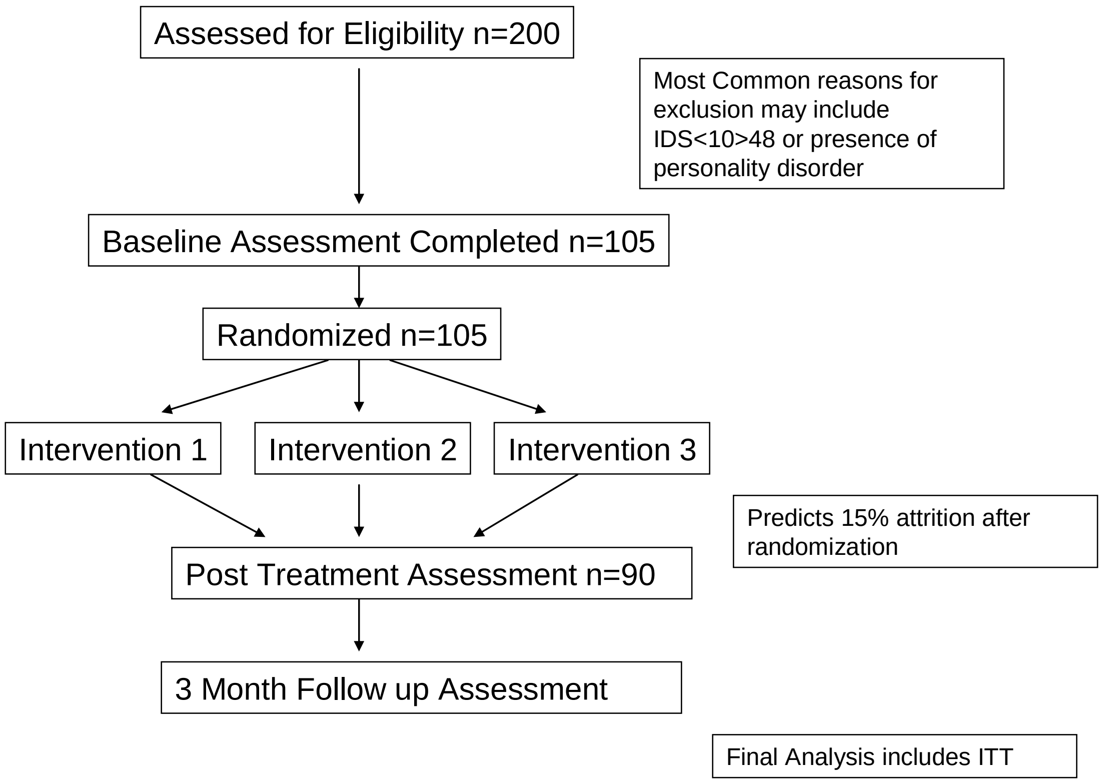

5.2 **Description of the Study Design**

This dismantling study will be a 3-armed controlled trial that compares the effects of attention/FA, acceptance/OM and MBCT (combined FA-OM) on clinical efficacy and three domains of PFC functioning in 105 individuals with mild-severe depression and high negative affect. The design is based on Jacobson's classic 3 armed dismantling study (Jacobson et al., 1996) which compares single elements of a treatment package to the full, combined package.

The number of study arms: 3
Name of Interventions: Mindfulness-Based Cognitive Therapy; focuses awareness meditation; open-monitoring meditation

Single or multi-center: single

Healthy or sick population: Individuals with mild-severe affective disturbances will comprise the sample because they represent the most frequent users of meditation-based therapies, and because depression and anxiety represent some of the world's largest health burdens (183-185) . This representative sample includes individuals who are both above and below the DSM-IV diagnostic threshold for major depressive disorder (MDD) and although not required, a past history of MDD and/or co-morbid anxiety symptoms are likely (37). Current psychiatric in-patients will not be eligible for the study.

Description of study groups/arms including sample size

The 90 anticipated completers will be nearly equally divided into each arm: FA, OM, and MBCT will each have approximately 30 participants. See flow chart.

Approximate time to complete study enrollment: 36 months

The expected duration of subject participation: 20 weeks (including follow-up)

A description of the sequence and duration of all trial periods, including follow-up (specify individual participants vs. entire trial)

For individual participants:

After screening, and successful completion of baseline assessments, groups of participants will assigned to either the 1.) focused-awareness, b.) open-monitoring, or c.) traditional mindfulness-based cognitive therapy arm. Each treatment lasts for eight weeks, followed by a post-treatment assessment, and 3-month follow-up interview.

Timeline overview of the entire award (years 1-5)

Preparation for this study will start in year 1-2, including treatment module development/refinement, training in assessments and participant recruitment. The original grant timeline allocated a minimum of 3.5 years (42 months) and a maximum of 4 years (48 months) to complete the treatment and data collection portion of the K-award. If the first treatment begins in the spring of 2013, then data collection is scheduled to be complete in Aug 2016 (42 months) or Dec 2016 (48 months).

The total target recruitment is 105 enrolled/90 completers. The number of groups and number of participants per group will depend on recruitment rates, but is estimated to be 9-12 groups of 8-12 enrolled (7-10 completer) participants. Each "group" requires a four month block of time that contains pre and post assessments and 2 months of treatment.

Thus, the plan for the next 3 cycles would be to run one group in the spring 2013, one in the summer 2013 and one in the fall of 2013. The number of completers for the first (spring 2013) group will be known by June 1st 2013.

Methods for collecting data for assessment of study objectives:

Data collection methods include clinical interviews, laboratory-based assessments, and self-report questionnaires (see Procedures Manual for details)

Primary and secondary outcomes to be measured during the trial

The primary objective is to asses and compare the effects of three types of meditation practices (FA, OM, MBCT) on emotional disturbance and wellbeing.

Secondary objective are 1) to assess and compare the effects of FA, OM and MBCT on subjective and objective measures of 3 domains of PFC functioning: attention, emotional reactivity/regulation and wakefulness and 2) To determine which neurobehavioral processes (attention, emotion regulation, wakefulness) drive the clinical benefits of mindfulness training, independent of treatment allocation or practice type.

Assessments:
All assessments will be made on all study participants regardless of study arm

There are several type of assessments:
Screening (see screening section and procedures manual
Clinical Interviews (pre-treatment and 3 month follow-up)
Self-report questionnaires (pre-post and during treatment, and 3 month follow up) Laboratory based assessment (pre-post treatment)

See Procedures Manual for details

5.3 Study Endpoints

5.3.1 Primary clinical Endpoint

Depression, negative affect and wellbeing:

	**Objective**	**Self-report**
Depression/negative affect	IDS interview	DASS
Positive affect/wellbeing		PANAS, WBS

Inventory of Depressive Symptomatology- Clinician Rated (IDS-C_{30}) (Rush et al., 1986; Rush et al., 1996)
The Depression, Anxiety Stress Scale (DASS) (Brown et al., 1997; Crawford & Henry, 2003; Lovibond & Lovibond, 1995)
Positive and Negative Affect Schedule – Expanded Form (PANAS-X) (Watson & Clark, 1994)
The Wellbeing Scale (WBS)(Ryff, 1989)

5.3.2 Secondary Endpoints

Attention, Emotion Regulation/Reactivity, and Wakefulness

**PFC Domain**	**Objective**	**Self-report**
attention	SART errors	FFMQ, MAAS
emotional reactivity/regulation	EMG response to emotional	DERS, FFMQ, SCO

	photos	
wakefulness	5-9 HZ EEG power<br>sleep latency	KSS

5.3.3 Exploratory endpoints

Factors that are not meditation type, that may influence outcome, including:

- therapist-level factors include warmth/empathy; adherence
- Participant level factors include personality/temperament

6. STUDY POPULATION

6.1 Overview

Human Subjects Involvement and Characteristics: Participants will complete a 6 month protocol including questionnaires, in lab assessments of attention, sleep propensity and emotion, 8 weeks of meditation training, and a 3 month follow-up interview.
105 participants will be enrolled from the general community ages (ages 18-65).
The sample was chosen to represent the population that most frequently uses meditation based modalities, (i.e. "stressed" individuals with persistent mild to severe levels of depression, anxiety and negative affect.)

6.2 Inclusion Criteria:

Participants must meet the following requirements to participate in the study

•English-speaking
•ages 18-65
•mild-severe levels of depression (IDS 10-48) or high level of negative affect (PANAS negative affect scale >20 in last month).
•May include the following DSM diagnoses and classifications of emotional disturbances:
296.xx Major Depressive Disorder, single episode or recurrent, chronic, or in partial remission;
300.4 Dysthymic Disorder;
311 Depressive Disorder NOS (minor depression and recurrent brief depressive disorder;
300.2 Generalized Anxiety Disorder;
293.84 Anxiety Disorder Due to a Medical Condition;
300.00 Anxiety Disorder NOS.

6.3 Exclusion Criteria:

Participants will be excluded if they meet any one of the following:
<u>At time of screening interview (past month)</u>
•Extremely severe levels of depression (IDS>48)
•active suicidal ideation (IDS-18 >2)

• presence of Axis I personality disorder
•panic disorder
•post-traumatic stress disorder
•obsessive-compulsive disorder
•eating disorder, or substance abuse/dependence
•inability to read and write in English,
•current psychotherapy (>1 mo)
•change in antidepressant medication type or dosage in the last 8 weeks.

Lifetime history exclusions:
• bipolar disorder
• psychotic disorders (cyclothymia, schizophrenia, schizoaffective disorder)
• persistent antisocial behavior or repeated self-harm,
•borderline personality disorder,
•organic brain damage
•regular meditation practice

Rationale: These participants are excluded because they may disrupt group participation, require additional or specialized treatment, are outside the range of meditation uses, are typically excluded from meditation studies and therefore not "representative", or may confound the results of the study.

6.4 Inclusion of Women and Minorities

Current census data indicate that the racial/ethnic breakdown of the state of Rhode Island is as follows: .3% of the population is American Indian or Alaskan Native, 1.0% is Asian, 4.5% is Black, 1.2% is Hawaiian/Pacific Islander, 8.4% is Hispanic/Latino, 80.7% is White, and 4% is some other ethnicity. While we expect that enrollment will represent these ratios, we will attempt to oversample minority participants to increase their representation in the sample to more closely match the racial and ethnic distribution of individuals living in the U.S. (75.1% White, 12.3% Black or African American, 3.6 % Asian, 0.9% American Indian/Alaska Native, 0.2% Native Hawaiian/Other Pacific Islander, 5.5% some other race, and 2.4% two or more races; 12.6% Hispanic/Latino and 87.4% Non-Hispanic/Latino. An effort will be made to enroll individuals of various ethnic and racial minority backgrounds by advertising in newspapers and clinics with a high minority clientele. Dr. Kohn, who is Director of the Multicultural Psychiatry Clinic at Butler, will consult on issues regarding the increased recruitment and retention of minority participants in the study.

Preliminary data suggest that females represent MBSR/MBCT study samples in a 3:2 ratio. Non-English speaking minorities will not be eligible for the study because the treatments will be delivered in English. Table 5 shows the number of study participants per ethnic/racial category to be enrolled based on Census data.

6.5 Inclusion of Children

Children under age 18 will not be eligible for the study because their inclusion would require age-appropriate adaptations to the treatments, and parental consent. Studies performed in our lab that include adolescents under 18 {Britton, 2010 #15} required substantially shorter meditation times than older samples. In addition, the prefrontal cortex, which is a central target of this study, is not fully developed in children.

6.6 Target/Planned Enrollment

Targeted/Planned Enrollment Table

Study Title: Dismantling Mindfulness: Contributions of Attention vs. Acceptance to the Treatment of Affective Disturbances

Total Planned Enrollment: 105

TARGETED/PLANNED ENROLLMENT: Number of Subjects			
**Ethnic Category**	**Sex/Gender**		
	**Females**	**Males**	**Total**
Hispanic or Latino	6	3	9
Not Hispanic or Latino	57	39	96
**Ethnic Category: Total of All Subjects ***	63	42	105
**Racial Categories**			
American Indian/Alaska Native	0	0	0
Asian	3	2	5
Native Hawaiian or Other Pacific Islander	1	1	2
Black or African American	5	3	8
White	54	36	90
**Racial Categories: Total of All Subjects ***	63	42	105

* The "Ethnic Category: Total of All Subjects" must be equal to the "Racial Categories: Total of All Subjects."

6.7 Co-enrollment Guidelines

Study participants may not co-enroll in any other treatment studies.

Enrollment in non-invasive assessment-only studies may be permitted, but must be approved by the PI.

6.8 Strategies for Recruitment and Retention

Recruitment: The Principle Investigator and research staff will recruit participants from the local community through a variety of sources: posting informational flyers/brochures, placing print advertisements, making personal appearances at community events, distributing study information through primary care clinics, and posting e-advertisements on relevant websites.

Retention:
A recent 2 armed RCT of MBCT vs waitlist indicated a retention rate of 93% for the waitlist group and 78% for the waitlist. Since all three arms of this study are active treatments, we project a retention rate similar to the MBCXT study (>85%) for all arms.

Several strategies will be used to increase retention, with the target goal of keeping drop outs below 15%. First, patients will be asked to provide the names and phone numbers of multiple individuals who always know how to contact the participant. Participants will receive calendars about treatment and study schedules and reminder calls before important dates. They will also be followed up if they miss a class or assessment. Retention procedures will be reviewed regularly with the mentorship team and revised accordingly

7. METHODS FOR MINIMIZING BIAS

7.1 Treatment Randomization

7.1.1 Background Rationale

Treatment randomization procedures have been discussed with mentors and are based on the following considerations:

7.1.2 Clinical and Treatment Considerations

- *Group size.* Ideal MBCT groups size = 8-12 (Segal); groups should not be smaller than 4
- *Wait time to treatment.* Minimal wait time between baseline assessment, randomization and beginning of treatment

7.1.3 Feasibility and practical considerations

- *Basic Calculations.* Because only one lab assessment can be done per day, with a reasonable goal of 1-4 per week, 4-16 subjects will be eligible for randomization in a given month. Given that an ideal class size is 8-12 (Max 16), with a minimum of 4, basic calculations yield enough participants for randomization into one treatment at a time,

with a range of 0-4 weeks wait time (Maximum 4 weeks) to enter treatment. This option would yield a minimal group size within 1 month, even if recruitment is extremely slow (1 per week). Alternatively, we would have to wait 3 months to get enough people to allocate into 3 simultaneous treatments, or have class sizes less than 4, which is unacceptable

- *Therapist and RA availability.* Since therapists are unpaid volunteers, they have agreed to run 1-2 groups per year each. Research Assistants are typically university students who operate on an academic year, and are less reliable during exams and holidays and transition periods.
- Based on previous research and as described in the Study Timeline section of the Research Strategy, we planned on 3 groups per year, 1 group each "season" (Fall, Spring, Summer) and gradually increasing our recruiting, assessment and therapist capabilities to 8-12 participants per group. Because of extended delays in the protocol review process, we expect to begin data collection about 12+ months behind schedule (Spring 2013).

7.1.4 Randomization Procedures

7.1.4.1 Introduction

The following randomization procedures have been adapted from 2 recent 3 armed MBCT trials by Zindel Segal, the creator of MBCT, and Jason Ong, another NCCAM K23 recipient. Dr. Segal is one of the consultants on this grant and has specifically recommend this procedure, which was also discussed and agreed upon my other mentors (Miller, Carskadon, Hirshberg) and biostatisticians (Kahler, Johnson, Seifer,)

•Segal, et al. (2010). Antidepressant monotherapy vs sequential pharmacotherapy and mindfulness-based cognitive therapy, or placebo, for relapse prophylaxis in recurrent depression. Arch Gen Psychiatry, 67(12), 1256-1264.

•Jason Ong. Augmenting Behavior Therapy for Insomnia with Mindfulness Meditation. NCCAM-funded K23 (K23 AT003678-01A1).

7.1.4.2 Timing:

Treatment programs are scheduled to start on Feb 15, June 1 and Sept 15 each year.
Randomization will occur no later than 3 days before the treatment start date. Randomization requires a quorum of at least 4 eligible participants who have completed baseline assessments. This will ensure adequate group size and minimize wait time to enter treatment.

7.1.4.3 Group Randomization

Treatment randomization is performed at the group level, rather than the individual level. For example, each group of 4-16 participants will be randomly allocated into one of three treatments (i.e. group randomization) 9 different times.

Stratification: Stratification by baseline characteristic will not be used because it would delay treatment, and because it is not possible in group randomization designs. Group differences in baseline characteristics will be assessed (see Preliminary Analyses section)

and if present, incorporated into analysis plan under the guidance of statistical mentors. Seasonal effects will be accounted for by balancing treatment equally over the 3 seasons (spring, Summer, fall).

Masking: The results of each treatment randomization will be recorded and communicated by an independent RA that is not involved in patient contact.

Procedure: An independent statistician, Tao Liu PhD., at Brown Center for Statistical Sciences is responsible for generating random treatment randomization sequences. The results of each randomization will be recorded by the statistician and communicated to the PI who is one of the co-therapists for each treatment. The PI will maintain the treatment location information in a password-protected electronic file, and provide de-identified codes to signify different treatments during analysis.

7.2 Blinding

Individuals who conduct post-treatment (8 week and 3 month) assessments will be blinded to treatment randomization. Since treatment randomization occurs after completion of baseline measures, there is no need for blinding at baseline.

All 3 interventions are active “mindfulness” meditation interventions (i.e. no control or placebo). The only study staff that will be unblinded are the PI and the other therapist who will be delivering the treatments.

7.3 Reliability

Assessments will be administered by an advanced RA blind to treatment condition. Dr. Miller and I will review assessment results in weekly meetings and supervise the assessors. Dr. Miller has successfully trained and utilized advanced RAs to conduct assessments in several previous NIH-funded clinical trials of hospitalized patients with severe mood disorders. All assessors will be trained to an initial inter-rater reliability (>.90) with periodic checks. A random sample of completed diagnostic and symptom assessments (15%) will be independently rated to determine the inter-rater reliability achieved.

8. STUDY INTERVENTIONS

MBCT is considered standard of care for treating active affective disturbances including clinically significant, mild to severe levels of depression (Barnhofer et al., 2009; Britton, Haynes et al., 2010; Eisendrath et al., 2008; Finucane et al., 2006; Kenny et al., 2007; Kingston et al., 2007; Kuyken et al., 2008; Schroevers et al., 2009; Shahar et al., in press; Williams et al., 2008) and anxiety (Evans et al., 2008; Finucane et al., 2006; Schroevers et al., 2009). In this study, the two main components of MBCT (focused awareness and open monitoring) will be offered as separate 8 week modules.

8.1 Treatment Matching

FA/OM and MBCT interventions are matched for duration, format and content.

Classes meet for 2.5 hours once a week for 8 weeks with a daylong retreat during the 6 or 7th week. Homework consists of 45 min/day of formal meditation practice.
Weeks 1-4 include practice instruction; 5-8 focus on applying the practices to manage acute negative affect.
See Appendix C for treatment manuals

8.2 Study Intervention Descriptions

8.2.1 Intervention #1: MBCT

MBCT (Segal, Teasdale, & Williams, 2004; Z. V. Segal et al., 2002; Teasdale, 2004) is an 8-week group-based intervention that combines principles from cognitive-behavioral therapy (Beck, Rush, Shaw, & Emery, 1979) and Mindfulness-Based Stress Reduction (MBSR; (Kabat-Zinn, 1990) using a psychoeducational and client-centered format. MBCT sessions focus on cultivating mindfulness or non-judgmental present-moment awareness of mental content and everyday activities, including sitting, lying down, breathing, walking, and other simple movements. Homework assignments consisted of practicing mindfulness meditation exercises with the aid of a guided audio CD and completing worksheets related to stress, automatic thoughts, and common reactions to various types of events. A session-by session description with handouts and homework assignments is available in the MBCT manual (Z. V. Segal et al., 2002).

8.2.2 Intervention #2: Focused Awareness

Focuses Awareness Mediataion (8 weeks). The FA module will include training in focused awareness techniques which require sustained attention on a selected object (or "anchor) with concomitant deselection of surrounding objects and events. Specific practices include the body scan meditation and standard variations of breath awareness from the MBCT/SR manuals and related sources (Santorelli et al., 2003; Sekida, 1985; Wallace, 2006; Williams et al., 2007).

8.2.3 Intervention #3: Open Monitoring

Open Monitoring Meditation (8 weeks): The OM module will include training in techniques that cultivate a non-judgmental *accepting relationship* to internal and external events, including thought and emotions, but without object selection or deselection. Specific practices include open monitoring ("choiceless awareness") meditation, mountain/ lake meditations, and labeling of mental states from the MBCT/SR manuals (Santorelli et al., 2003; Segal, Williams et al., 2002; Williams et al., 2007).

8.3 Therapist qualifications and training

8.3.1 Qualifications:

All instructors have more than 10 years experience with mindfulness meditation, and at least 5 years of teaching experience.

8.3.2 Training

All instructors co-taught meditation courses with the PI and received feedback from both the PI and the participants on the clarity of their instructions and impact of the different practices.

8.4 Therapist Competency, Treatment Fidelity and Adherence

Creation of treatment manuals for each module is part of the K-award and will be supervised by Drs. Segal, Roth, Jha, and Miller and other members of the Mindfulness Research Working Group. All sessions will be tape-recorded. Measures of treatment integrity will be based on multiple ratings of adherence/divergence from the treatment criteria (Jacobson et al., 1996; Segal, Teasdale et al., 2002).

8.4.1 Adherence rating by trained judges:

Adherence will be based on variations of the MBCT adherence scale (Z V Segal et al., 2002) Competency (Crane, Kuyken, Hastings, Rothwell, & Williams, 2010), will based on variations of the Mindfulness-Based Interventions Teaching Assessment Criteria (MBI-TAC) (Crane et al., 2011).

8.4.2 Ratings by Participants:

In addition to ratings from trained judges, therapists will be rated by participants according to the ***Therapeutic Factors Inventory*** (Lese & MacNair-Semands, 1996) ,***The Working Alliance Inventory*** (WAI; Horvath & Greenberg, 1989) and ***The Empathy Scale*** (ES; Burns & Auerbach, 1996)

8.5 Participant Compliance with Intervention(s)

Class Attendance
Homework Compliance:
Meditation practice logs: Participants keep track of their daily meditation practice during the 8 weeks of active treatment and follow up periods. Diaries include information about meditation practice including: a) the type of meditation (body scan, breath awareness, etc.), b) the number of minutes practiced, c) whether they fell asleep during practice d) use of CD/tape; and informal practice (walking, mindful activities). Logs for the preceding week are collected at each class meeting.

Mindfulness Skill Acquisition: The following inventories will be used to assess the degree to which the participants have acquired various mindfulness skills:

•Five Factors of Mindfulness Questionnaire (FFMQ) (Baer et al., 2006)
•The Mindful Attention Awareness Scale (MAAS) (Brown and Ryan 2004
•Mindfulness Skill Acquisition(Britton & Shahar, 2003)

9. STUDY SCHEDULE

See also Procedures Manual for Details.

9.1 Screening

Screening is a two step process and includes a phone screen and in-person interview.

9.1.1 Phone screen

exclusion criteria include:

<u>Current:</u>

•age <18 or >65
•inability to read and write in English

<u>Lifetime history of (known or diagnosed):</u>

• bipolar disorder
• psychotic disorders (cyclothymia, schizophrenia, schizoaffective disorder)
• persistent antisocial behavior or repeated self-harm,
•borderline personality disorder,
•organic brain damage
•regular meditation practice

9.1.2 In person interview

exclusion criteria include:

<u>At time of screening interview (past month)</u>

•Extremely severe levels of depression (IDS>48)
•PANAS NA <18
•active suicidal ideation (IDS-18 >2)
•presence of Axis I personality disorder
•panic disorder
•post-traumatic stress disorder
•obsessive-compulsive disorder
•eating disorder, or substance abuse/dependence
•inability to read and write in English,
•current psychotherapy (>1 mo)
•change in antidepressant medication type or dosage in the last 8 weeks

<u>Lifetime history exclusions:</u>

• bipolar disorder
• psychotic disorders (cyclothymia, schizophrenia, schizoaffective disorder)
• persistent antisocial behavior or repeated self-harm,
•borderline personality disorder
•organic brain damage

9.1.2.1 Concomitant Medications and Procedures

All medication are permitted as long as there is no change within 2 months of baseline assessment or during the active phase of the study.

If the medication is psychotropic (i.e anti-depressants), changes in type or dose is prohibited duration the study. If a change in medication is warranted for safety reasons, the participant will be allowed to finish the treatment but will be considered non-compliant with protocol (i.e data not included in analysis).

Participants who are not stabilized on their (psychotropic) medications for at least 2 months may enroll later, when they have stabilized.

9.1.2.2 Provisional inclusions:

Current psychotherapy: If a patient is currently participating in psychotherapy in the community, the potential subject will be referred back to the outpatient provider(s) to fully discuss the pros and cons of terminating treatment for purposes of study enrollment.
Recent medication change: Subjects who changed their psychotropic medication in the last 8 weeks, but are otherwise eligible may enroll pending another SCID interview when they have stabilized on their medications.
Current panic, PTSD, OCD: Patients may return for assessment if these conditions are successfully treated or spontaneously remit.

9.1.3 Exclusion Procedures:

If the subject does not meet entry criteria, and is unlikely to in the future, they may receive a list of local providers or and/or other possible treatment studies.

Active suicidal ideation: If the participant is excluded because of active suicidal ideation, follow the suicide assessment procedure in the "Managing Suicidality" manual.

9.2 **Informed Consent**

If the subject meets entry criteria,**, they will undergo the following informed consent procedures:**
1) Briefly, describe the study, including:
•Timeline and procedure
•Risk and benefits and alternative treatments
• **the** decision to not participate or to withdraw from the study will not adversely affect current or future interactions with the Neurodevelopment Center or Brown University.

2) Provide a copy of the IRB-approved consent form and allow them to read and ask questions.
3) Then quiz the subject via open-ended questions about important aspects of the study protocol to assess his/her understanding of study procedures. For example, “What kind of treatment

would you receive if you take part in this study?" Acceptable answer: "Mindfulness Meditation training for 8 weeks." If the patient answers incorrectly, the interviewer will review the relevant section of the consent form again and query the patient until (s)he can provide an appropriate response, in a process called iterative feedback.

9.3 Baseline Assessment

Subjects complete baseline questionnaires and laboratory assessments at the Neurodevelopment Center. See Procedures Manual for Details.

9.4 Enrollment

Formal enrollment in the study requires the following:
1) Meet all entry criteria after screening
2) Completed informed consent procedures
3) Completed all baseline assessments (both self-report and laboratory assessment)
4) Continued interest and availability to continue the study after completing #1-3
5) Completion of treatment randomization procedures

9.5 Treatment Randomization

Within 1 month of the first completed baseline assessment, if at least 3 other participants have completed assessment (total of 4), proceed to randomization to choose a treatment. See Treatment Randomization for procedure.

9.6 Treatment

Participants enter one of three active treatments for 8 weeks. See Treatment Section. And treatment manuals

9.7 Post Treatment Assessment

After the 8th class, participants repeat the baseline assessment procedures (sleep diaries, z-machines, self-report questionnaires and laboratory assessments).

In addition to outcome questionnaires, participants will also complete questionnaire to their experience of the therapist and the treatment experience. See section on Therapist Adherence

9.8 3 Month Follow-up Assessment

Three months after the post-treatment assessment, the participants returns for an in-person clinical assessment interview (IDS, PANAS, DASS, WBS). The participant will also complete a

3rd set of self-report questionnaires. For details, see Table 2.1 Timeline and Summary of Assessments in the Procedures Manual.

9.9 Adverse effects

Patients who exhibit clinical deterioration (an increase in IDS score of > 16 points) or Non-responders (identified as minimal change in symptoms from baseline assessment) will be assessed for safety and provided with appropriate clinical follow-up, including referrals to other treatment, if desired.

Patients who express active suicidal ideation (IDS 18=3) will be followed with the suicidaility management procedure (described in detail in the Procedures Manual Safety Section) and monitored until resolution.

9.9.1 Stopping Rules for an Individual Participant

A study participant will be discontinued from further Study Intervention(s) if:

- Any clinical adverse event, laboratory abnormality, intercurrent illness, other medical condition or situation occurs such that continued participation in the study would not be in the best interest of the participant.

Development of any exclusion criteria may be cause for discontinuation.

Participant will continue to be followed with participant’s permission if Study Intervention is discontinued.

10. ASSESSMENT OF SAFETY

10.1 Ethics/Protection of Human Subjects

10.1.1 Institutional Review Board/Ethics Committee

The above protocol is subject to review and approval by the Brown University Institutional Review Board (IRB) prior to the implementation of the protocol. Any amendments to the protocol or consent materials must also be approved before they are placed into use.

10.1.2 Informed Consent Process:

Written informed consent will be obtained from each subject at entry into the study. Informed consent is obtained by the following process:

1. The subject will be asked to review the study consent form.
2. The PI or Co-Investigator (Co-I) will meet with the subject to review the form, to confirm the subject's understanding of the study, and to answer any questions that the subject might have.
3. Once the subject demonstrates understanding of the study and agrees to participate in the study, the consent will be signed in the presence of eligible study staff.

10.1.3 Adherence

The Study Protocol for the **K23 AT006328-01A1** will adhere to the protocol approved by the Brown University IRB. Major modifications to the IRB protocol that are considered a change in scope, the scientific intent, study design, patient risk, or human subject protection (i.e. change in control group) will be discussed with appropriate NCCAM officers prior to implementation. Minor modifications that are considered editorial or administrative updates (i.e. address changes) do not need prior approval "as long as the change does *not* affect the scientific intent, study design, patient risk, or human subject protection" (NCCAM 2012, Guidance on Changes in Clinical Studies in Active Awards – When is Prior NCCAM Approval Required?)

10.1.4 Attached IRB Documents:

The following documents are included in the Regulatory Binder.

- Brown IRB approval
- IRB-approved Consent Form

10.2 ADVERSE EVENTS

10.2.1 Definition of an Adverse Event (AE)

"An adverse event (AE) is any untoward medical occurrence in a subject temporally associated with participation in the clinical study or with use of the experimental agent being studied. An adverse finding can include a sign, symptom, abnormal assessment (laboratory test value, vital signs, electrocardiogram finding, etc.), or any combination of these."

10.2.2 Definition of a Serious Adverse Event (SAE)

Serious Adverse Event (SAE): A Serious Adverse Event is defined as an AE meeting one of the following conditions:

- Death
- A life-threatening event
- Inpatient hospitalization or prolongation of existing hospitalization
- A persistent or significant disability/incapacity
- A congenital anomaly or birth defect

- Important medical event based upon appropriate medical judgment"

10.2.2.1 AE/SAE Severity

- ***Grade 1 (Mild)****: events require minimal or no treatment and do not interfere with the patient's daily activities.*

- ***Grade 2 (Moderate)****: events result in minor inconvenience or concern with the therapeutic measures. Moderate events may cause some interference with functioning.*

- ***Grade 3 (Severe)****: events interrupt a patient's usual daily activity and may require systemic drug therapy or other treatment. Severe events are usually incapacitating.*

- ***Grade 4 (Life threatening)****: Any adverse drug experience that places the patient or participant, in the view of the investigator, at immediate risk of death from the reaction as it occurred, i.e., it does not include a reaction that had it occurred in a more severe form, might have caused death.*

- ***Grade 5 (Death)***

10.2.2.2 Relationship of AEs/SAEs to the Study Intervention

This designation will be made by the principal investigator.

AEs will be categorized according to the likelihood that they are related to the study intervention. Specifically, they will be labeled definitely unrelated, definitely related, probably related, or possibly related to the study intervention.

Definitely Related
There is clear evidence to suggest a causal relationship, and other possible contributing factors can be ruled out. The clinical event, including an abnormal laboratory test result, occurs in a plausible time relationship to study agent/intervention administration and cannot be explained by concurrent disease or other drugs or chemicals. The response to withdrawal of the study intervention should be clinically plausible. The event must be phenomenologically definitive, with use of a satisfactory rechallenge procedure if necessary.

Probably Related
There is evidence to suggest a causal relationship, and the influence of other factors is unlikely. The clinical event, including an abnormal laboratory test result, occurs within a reasonable time sequence to administration of the study agent/intervention, is unlikely to be attributed to concurrent disease or other drugs or chemicals, and follows a clinically reasonable response on withdrawal (dechallenge). Rechallenge information is not required to fulfill this definition.

Possibly Related

There is some evidence to suggest a causal relationship (e.g., the event occurred within a reasonable time after administration of the trial medication). However, the influence of other factors may have contributed to the event (e.g., the subject's clinical condition, other concomitant events). Although an adverse drug event may rate only as "possible" soon after discovery, it can be flagged as requiring more information and later be upgraded to probable or certain as appropriate.

Unlikely
A clinical event, including an abnormal laboratory test result, whose temporal relationship to study agent/intervention administration makes a causal relationship improbable (e.g., the event did not occur within a reasonable time after administration of the trial medication) and in which other drugs or chemicals or underlying disease provides plausible explanations (e.g., the subject's clinical condition, other concomitant treatments).

Not related
The AE is completely independent of study study agent/intervention administration, and/or evidence exists that the event is definitely related to another etiology. There must be an alternative, definitive etiology documented by the clinician Expected Events Related to Disease Process: Provide explicit definitions of the type(s), grade(s), and duration(s) of adverse event(s) that will be considered disease related.

10.2.2.3 Expected Risks -NCCAM statement

The following is a statement from NCCAM about the safety profile of meditation practice:
Side Effects and Risks of Meditation

Meditation is considered to be safe for healthy people. There have been rare reports that meditation could cause or worsen symptoms in people who have certain psychiatric problems, but this question has not been fully researched. People with physical limitations may not be able to participate in certain meditative practices involving physical movement. Individuals with existing mental or physical health conditions should speak with their health care providers prior to starting a meditative practice and make their meditation instructor aware of their condition.

SOURCE: http://nccam.nih.gov/health/meditation/overview.htm#sideeffects (Oct 26,2011)

Expected risks to the subject are as follows:

Discomfort during meditation: Meditation-based interventions may results in discomfort with attention to unpleasant thoughts, feelings or body sensations. Some individuals may experience an initial increase in undesirable emotions with increased attention to them.
Discomfort during assessments: Some participants may feel uncomfortable answering personal questions about their psychological health. The physiological recording electrodes may cause mild skin irritation. Some of the stimuli may be unpleasant or graphic.

Risk of adverse events during the study. It is possible that some patients will have an adverse event during the study, including failure to respond to treatment, worsening of depressive symptoms, suicidal ideation or behavior, or other adverse events.

10.2.2.4 Impact statement
:
"These risks are considered to be minimal and are addressed in the protocol and consent form."

10.2.2.5 Measures that will be taken to minimize study risk:

Meditation-related discomfort: Regarding psychological discomfort associated with mindfulness practice, participants are self-guided and are encouraged to set their own limitations. In addition, the curriculum of the training program is specifically geared towards dealing with such discomfort.

Assessment-related discomfort: None of the assessment procedures pose a serious risk to volunteers. With regard to the collection of polysomnographic data, procedures that are standard in Dr. Richard Bootzin's Sleep Research Laboratory will be used. These procedures have been used with hundreds of participants, with no reports of death or injury attributed to the procedures. Dr. Bootzin's group has reported minor skin irritations from tape placed on the face in a minority of cases. For the EEG/EMG recordings, precautions such as using latex-free or micropore tape and conductant paste (instead of glue) to affix electrodes will be taken.

The clinician rating and self report measures are commonly used for research and clinical purposes. There are no known risks attendant to its administration. Well-documented procedures for eliciting emotion are used, there are no known risks associated with these procedures.

4) Adverse events:

Clinical Deterioration and Suicidality: Participants assigned to any treatment group may experience significant clinical deterioration or suicidal ideation. All patients will be monitored by study staff at weekly intervals in person or by telephone and assessed every two weeks with questionnaires about mood and affect. Dr. Miller and Dr. Hirshberg are licensed clinical psychologists and have extensive experience evaluating research participants for clinical deterioration or suicidality. Significant risk for suicide will be defined by an IDS item 18 score = 3, or the report of a desire or intent to hurt oneself to any member of the study staff. At follow-up, study staff may also identify participants with significant clinical deterioration (as defined by an IDS score > 40 (which is equivalent to a 17-item Hamilton Rating Scale for Depression (HRSD) score > 20) or an increase in IDS score of > 16 points, which is equivalent to an increase in HRSD score of > 8 points. If study staff identify an individual with significant clinical deterioration or who report any suicidal ideation, we will utilize our standard protocol for managing these problems. Study staff will immediately contact Dr. Britton or Miller, who will evaluate the patient over the telephone or in person. First, they will conduct a suicide risk assessment to determine whether it is necessary to take immediate action to prevent the participant from causing harm to herself. If needed, actions that Drs. Britton or Miller may take include having a family member bring the person to Butler Hospital or sending an ambulance so that the individual may be evaluated for inpatient psychiatric admission. For detailed procedures,

please see Procedures Manual: *Manual for Managing Suicidal Ideation and Behavior* and *Psychiatric Hospitalization Procedures*. If the study participant has experienced significant deterioration, but is not in immediate danger of hurting herself, we will take the following actions. First, we will inform the patient about procedures for contacting emergency services should they find themselves at risk for self-harm. Second, with the patient's knowledge, we will contact their primary care provider to inform them of the deterioration. We will urge the patient to make an appointment with that provider. Third, if the patient consents, we will speak with one of their family members to ensure that she is aware of the seriousness of the patient's symptoms and the agreed-upon treatment plan. We will provide psychotherapy referrals if the patient wishes them. Participants who a) require an inpatient psychiatric admission; or b) begin antidepressants or psychotherapy while in the study will be officially discontinued from the study at that point. We will collect endpoint data at the point of discontinuation. Depending on reasons for discontinuation and our clinical judgment, participants may be allowed to finish out their time in the meditation class.

Non-Response to Treatment: Independent of clinical deterioration, the possibility that the treatment will not yield benefit is another possible risk and will be explained during informed consent procedures. All patients will be monitored by study staff in person or by telephone at weekly intervals and assessed every two weeks with questionnaires about mood and affect. Non-responders (identified as minimal change in symptoms from baseline assessment) will be provided with referrals to other treatment, if desired.

10.3 Data Collection Procedures for Adverse Events

10.3.1 Scheduled collection

(IDS interviews):
Item 18 =3 (suicidal ideation)
IDS total has increased >16 points since the last assessment
OR IDS total score is > 48

Meditation logs:
Collected at weekly sessions.
Comments sections contain possible adverse events.
Participants contacted for clarification and follow up.

Direct observation during sessions (weekly).
Study staff will have direct contact with participants.
Will query participants who may be having AEs

3-month follow up;
Meditation Experiences Invereview (MEDEX-I)

Participants queried about potentially distressing meditation experiences by independent asssessor

10.3.2 Unscheduled collection

Participants are encouraged to contact the PI or instructor if they have any questions or problems in regard to meditation practice, their mental status or study participation.

10.4 Recording/Documentation

10.4.1 AE Reporting and Follow-Up

Adverse events and study progress will be monitored and recorded on an ongoing basis. We will use the following suggested table for reporting subject status and adverse events:

Subject Status

Pt Identifier	Date Enrolled	Date Completed Study	Study Status	Reason for Withdrawal	% Adherence to Intervention	Intervention Duration (Weeks)
Subj001	08/02/2010	N/A	A			
Subj002	07/26/2010	9/20/2010	C		87%	8 weeks
Subj003	08/04/2010	N/A	W	Injury unrelated to intervention		

<u>Status:</u>
A = Active
C = Completed
W = Withdrew
L = Lost to Follow Up

<u>% Compliance to Intervention:</u>
% classes attended (8=100%)

Adverse Events Reporting Table

Pt Identifier	AE Onset	AE End	AE Code (MedRA, CTCAE)	Severity	SAE? (Y/N)	Relatedness	Action Taken	Outcome	Comments
Subj001	08/02/2010			1		2	1	4	Subj felt Achilles tendon pain during yoga class; history of tendonitis; will not attend class next week
Subj002	07/26/2010			10/02/10		C			

Subj003	08/04/2010			3		0	4	4	Subj broke leg while skiing; withdrew from study

Severity of AE:
1 = Mild
2 = Moderate
3 = Severe
4 = Life threatening or disabling

Relatedness to Intervention:
0 = Definitely unrelated
1 = Unlikely
2 = Possibly Related
3 = Probably Related
4 = Definitely Related

Action Taken:
0 = None
1 = Dose modification
2 = Medical intervention
- (specify in comments)
3 = Hospitalization
4 = Intervention discontinued
5 = Other

Outcome:
1 = Resolved
2 = Recovered with minor sequelae
3 = Recovered with major sequelae
4 = Continuing treatment
5 = Condition worsening
6 = Patient death**

10.4.2 Reporting Procedures

10.4.2.1 SAEs

SAEs that are unanticipated, serious, and possibly related to the study intervention will be reported to the Independent Monitor(s), IRB, and NCCAM in accordance with requirements. Unexpected fatal or life-threatening AEs related to the intervention will be reported to the NCCAM Program Officer within 7 days.
Other serious and unexpected AEs related to the intervention will be reported to the NCCAM Program Official within 15 days. Anticipated or unrelated SAEs will be handled in a less urgent manner but will be reported to the Independent Monitor(s), IRB, NCCAM, and other oversight organizations in accordance with their requirements. In the annual AE summary, the Independent Monitor(s) Report will state that they have reviewed all AE reports.

10.4.2.2 Changes in Study Status

During the funding of this study, any action of an IRB or one of the study investigators that results in a temporary or permanent suspension of the study will be reported to the NCCAM Program Official within 1 business day of notification.

10.5 Stopping Rules for the Protocol

This study will be stopped prior to its completion if: (1) the intervention is associated with adverse effects that call into question the safety of the intervention; (2) difficulty in study

recruitment or retention will significantly impact the ability to evaluate the study endpoints; (3) any new information becomes available during the trial that necessitates stopping the trial; or (4) other situations occur that might warrant stopping the trial.

Specifically, the occurrence of multiple severe adverse events that were definitely or probably related to meditation, in the entire intervention group, or a subgroup -would trigger an ad hoc safety review.

Enrollment and/or study intervention(s) would be suspended until a safety review is convened The objective of the safety is a decision as to whether the study agent/intervention (for an individual or study cohort) should continue per protocol, proceed with caution, be further investigated, be discontinued, or be modified and then proceed.

10.5.1 Stopping Rules for an Individual Participant

A study participant will be discontinued from further Study Intervention(s) if:

- Any clinical adverse event, laboratory abnormality, intercurrent illness, other medical condition or situation occurs such that continued participation in the study would not be in the best interest of the participant.

Development of any exclusion criteria may be cause for discontinuation.

Participant will continue to be followed with participant's permission if Study Intervention is discontinued.

10.6 DSMB Independent Monitoring Committee

10.6.1 Members of Monitoring Committee

The Monitoring Committee for this study is comprised of:

Drs. David Fresco, Ph.D. (Kent State), is a clinical psychologist who conducts clinical trials in mindfulness interventions.
Gary Epstein-Lubow. M.D. (Brown University Medical School) is a psychiatrist with special training in mood disorders, and has some experience with mindfulness and meditation-based treatments.
Jennifer Johnson, PhD is a biostatistician who has provided data analysis for many published studies, including methodological studies and studies requiring advanced methods such as multilevel structuralequation modeling, Cox regression with time-varying covariates, general estimating equations, and zero-inflated negative binomial regression (see CV publications 2-10, 12, 13, 19-21, 26-28, 31, 34) and serve as the study statistician on several grants (R34 MH094188, R34 DA030428, R34 MH086682, and K23 DA021159). In particular, because of

her background in group psychotherapy research, she is expert in methods of grouped data analysis and analysis of group-randomized trials, and wrote a paper (publication #2) illustrating the application of multilevel structural equation modeling to group psychotherapy research.

Drs. Fresco, Johnson, and Lubow are not associated with this research project and thus work independently of the PI, Dr. Willoughby Britton. They are not part of the key personnel involved in this grant. They are qualified to review the patient safety data generated by this study because of their unique expertise in the area of mindfulness interventions, anxiety/depression, stress, and mood disorders. The CVs of all members of the IMC are attached in Appendix D.

10.6.2 Safety Review Plan

Study progress and safety will be reviewed quarterly (and more frequently if needed). Progress reports, including patient recruitment, retention/attrition, and AEs will be provided to the Independent Monitor(s) following each of the quarterly reviews. An Annual Report will be compiled and will include a list and summary of AEs. In addition, the Annual Report will address:
(1) whether AE rates are consistent with pre-study assumptions;
(2) reason for dropouts from the study;
(3) whether all participants met entry criteria;
(4) whether continuation of the study is justified on the basis that additional data are needed to accomplish the stated aims of the study; and
(5) conditions whereby the study might be terminated prematurely.

The Annual Report will be sent to the Independent Monitor(s) and will be forwarded to the IRB and NCCAM. The Brown IRB will review progress of this study on an annual basis.

10.6.3 Subject Accrual and Compliance

10.6.3.1 Measurement and Reporting of Subject Accrual, Compliance with Inclusion/Exclusion Criteria:

Review of the rate of subject accrual and compliance with inclusion/exclusion criteria will occur every 3 months to ensure that a sufficient number of participants are being enrolled and that they meet eligibility criteria and the targeted ethnic diversity goals outlined in the grant proposal (Targeted/Planned Enrollment Table). The tables provided will be used for data collection.

10.6.3.2 Measurement and Reporting of Participant Adherence to Treatment Protocol

Data on adherence to the treatment protocol will be collected weekly by research staff and reviewed every 3 months by the PI, the study statistician, and the IMC. Adherence of participants will be evaluated by class attendance and by monitoring daily meditation

practice homework. The suggested dose is 45 min/day 6 days week (=270 min/week= 100%) with typical adherence for this population around 70%. Rates of adherence lower than 70% are an outcome of interest, as they suggest a differential preference for certain types of practice. If adherence falls below the typical rate of 40%, study investigators and the IMC will discuss ways to capture the reasons behind adherence differences and whether ways to improve adherence should be implemented.

10.6.4 Frequency of Data Review for this Study

The DSMB will hold at least two meetings a year with personnel in Boston and Providence meeting in person and other members outside joining by teleconference. At these meetings, the DSMB will meet first in open session attended by the study investigators and then in closed session when reviewing emerging trial data. In addition, the board will be in contact at least once a quarter in order to carry out the functions of the DSMB such as monitoring enrollment and study retention rates, reviewing patient safety, risks of participation, and protocol adherence. The DSMB will inform the Brown IRB of its findings. The aims of the study will be re-evaluated at least once a year; appropriate modifications will be made in consultation with NCCAM, if necessary.
The frequency of data review for this study differs according to the type of data and can be summarized in the following table,

**Frequency of safety review by data type**		
**Data type**	**Frequency of review**	**Reviewer**
Subject accrual (including compliance with protocol enrollment criteria)	quarterly	PI, Independent Monitor(s)
Status of all enrolled subjects, as of date of reporting	quarterly	PI, Independent Monitor (s)
Adherence data regarding study visits and intervention	quarterly	PI, Independent Monitor (s)
AEs and rates	quarterly	PI, Independent Monitor (s)
SAEs	Per occurrence	PI, Independent Monitor (s) NIH/NCCAM

10.6.4.1 Study Report Outline for the Independent Monitors(s) (Interim or Annual Reports)

The Study Reports for the Independent Monitor(s) will follow the format provided in the sample document (enrollment, retention, demographics, AEs, SAes etc). The report will not provide data on primary or secondary endpoints, and tables will be generated only from aggregate (not by group assignment) data for the study population.

11. STATISTICAL CONSIDERATIONS

11.1 Study overview:

The research study is part of a Career Development Award. The purpose of the research is to provide experience and training in clinical trials methodology and provide pilot data to inform a R01 application.

The trial is a 3-armed "dismantling" design, in which the standard treatment package (MBCT) is compared with individual 2 single-component treatment of the same duration. Due to the budgetary constraints of this K23 award and the pilot nature of the project, the primary objective will be to estimate treatment parameters (in contrast to absolute significance levels) for single practice modules relative to the standard package to inform a larger clinical trial [PAR-03-078].

11.2 Objectives and Hypotheses:

The analysis is divided into 3 categories:

Preliminary Analysis includes assessment of delivery and acceptability of treatments, and any group inequalities that may have resulted from treatment randomization, attrition and compliance

Primary Analysis assesses: Effect of treatments on clinical outcome (negative affect/depression) and (secondary analyses) effect of treatment on mechanism of action.

Differences in clinical outcomes between active treatments tend to be small. Thus, given our sample size, we do not predict a significant difference between single-component the standard package in terms of clinical efficacy. However, because single practice interventions are less complex and easier to implement, non-inferiority to standard treatment has significant clinical meaning.

The largest group differences are hypothesized in terms of mechanism of action. Specifically, we predict:
Attention training (FA) will differentially improve attention (FA>MBCT>OM)
Acceptance training (OM) will differentially improve emotional non-reactivity (OM>MBCT>FA).

11.3 Analysis Plan:

11.3.1 Preliminary Analyses:

The purpose of the preliminary analysis is to investigate the data for patterns that may impact subsequent analyses, such as: group differences at baseline, differential attrition, compliance or instructor qualities, competence, adherence or ratings. Because we used a group randomization, we must also assess the degree to which group-ness influences outcome (i.e. intra-group correlation).

11.3.2 Primary Analyses:

To asses and compare the effects of FA and OM modules on clinical efficacy vs standard MBCT Multilevel Modeling (MLM) will be used to compare the trajectories of clinical improvements in affective states (IDS, DASS PANAS, WBS) for each treatment.

Level 1 variables will include depression, negative affect and wellbeing scores at multiple time points.
Level 2 variables will include treatment type, meditation practice amount and demographic variables (age, sex, education). A MLM approach (Bryk & Raudenbush, 1992) was chosen for analysis because it is well suited to repeated measures designs, yields more information than ANOVAs (i.e. allows for individual growth curves), has the ability to retain missing cases, and fits my training goals of advanced statistical analysis skills for treatment research.

11.3.3 Secondary Analysis:

11.3.3.1 To compare the effects of FA, OM and MBCT on attention, emotion regulation and wakefulness:

A linear mixed model approach will be used to compare the effects of each treatment on each measure.

Type of treatment and time (baseline vs. post-intervention) will be modeled as fixed effects. In addition, variables identified in preliminary analysis will be added to the model as predictor variables or covariates.

Description of outcome variables:
Attention: Changes in total SART errors (omission/commission)

Emotional Reactivity: Corrugator response (log10 mV^2 for the 45–200-Hz EMG band) during first 4s of picture presentation (i.e. before meditation instruction) will be used to assess emotional reactivity to unpleasant relative to neutral pictures. This comparison reflects the traitlike effects of meditation training in the absence of deliberate affect regulation efforts.

Emotion Regulation: The 4-8s post picture onset follows FA or OM meditation instruction and therefore represents the acute affect regulation effects of *active* meditation. Emotion regulation magnitude is defined as the decrease in corrugator EMG power during 4s of meditation vs initial 4s (unregulated) response. All subjects receive both FA and OM instructions, but only the practice that corresponds to the treatment module will be used in analysis (i.e. FA-based regulation for the FA group etc). The average of the combined FA and OM-based regulation will be used for MBCT group.

Wakefulness/Sleepiness: Computer-assisted spectral analysis based EEG measurements of sleepiness will be measured by the mean log transformed power spectral density (mV^2) of 5-9 Hz band at F3/A2, F4/A1 sites for 4 minutes. Neurobehavioral measurement of sleepiness (minutes until sleep onset) will be based on real-time visual analysis of PSG data, including EEG, eye movements, EMG (chin) tone.

11.3.3.2 To determine which neurobehavioral process drives the clinical benefits of mindfulness:

I chose multiple regression because it can evaluate associations between clinical outcome and multiple correlated continuous and categorical variables (it also fits my training goal of increasing multivariate statistical knowledge). Multiple regression analyses will be conducted across all groups to maximize power and allow for between-treatment comparisons. Criterion variables will include clinical outcome measures of affective disturbance and wellbeing (IDS, DASS, PANAS, WBS) (baseline- 3 month follow-up change scores). Aggregate scores for 1) attention, 2) emotional reactivity/regulation and 3) wakefulness will be entered as separate predictors. Variables that are not equivalent at baseline but may impact outcomes (e.g. sex, education etc) will also be entered as predictor variables. Hypothesized interactions between wakefulness, attention and emotion regulation will be also evaluated using multiple regression.

11.4 Sample Size Considerations

Sample Size and Power Estimation:

11.4.1 Objectives

The K23 study is intended to provide pilot data to support an R01 application for a larger fully-powered RCT. In a staged model of treatment development [PA-03-066], a pilot study is where "data are gathered to estimate intervention parameters (e.g., effect size, attrition, rates, response rates) and to perform preliminary power analyses" [NIMH PAR-03-078]. Therefore, the proposed project will estimate treatment parameters for FA and OM modules relative to MBCT to inform future larger-scale trials, not necessarily to determine absolute significance levels. A larger R01 trial will be needed for a definitive test to detect if true differences exist between treatments.

11.4.2 Feasibility limitations – maximum capacity

Based on a maximum of 9-12 groups of 8-12 participants, our maximum capabilities are about approximately 90-110 (enrolled) participants.

11.4.3 Relevant pilot data:

For our lab's MBCT trial (n=45), which consisted of a very similar population, intervention and equipment to the proposed study, effect sizes (Cohen's d) for MBCT vs. Waitlist Control) were
.89 for depression (BDI) (large)
.72 for decrease in SWS (large)
.75 for decreased emotional reactivity (i.e. large).

11.4.4 Basic Calculations

While effect sizes in mood and sleep following mindfulness training are large, the differences between active treatments tend to be small (Wampold et al., 1997).
A sample size of 90 (30 per intervention) will be able detect a small effect size (d=.33, power >.80, alpha=.05).

Method of calculation: G*power 3.1.3

F tests - ANOVA: Repeated measures, within-between interaction

**Analysis:**	A priori: Compute required sample size		
	α err prob	=	0.05
	Power (1-β err prob)	=	0.80
	Number of groups	=	3
	Number of measurements	=	2
	Nonsphericity correction ε	=	1
**Output**			
	**Total sample size = 90**		
	**Effect size d =.335**		

11.4.5 Additional Sample size considerations +modifications

11.4.5.1 Attrition

Based on pilot studies, we estimated 15% attrition rate (Shahar et al., in press) so that we need to enroll a total of 105 subjects (35 per group) in order to have 90 completers.

11.4.5.2 Sample size adjustment due to group randomization

Group randomization may require an increase in sample size to maintain power, if the responses of subjects in same group are correlated (intragroup correlation). However, if intra- group correlation = 0, then there is no need for adjustment (Friedman et al., 2010)

In a set of MBCT RCTs that used group randomization procedures and multilevel modeling analysis, Williams (2008) estimated IGCs for depression related outcomes (BDI, relapse rate). Most IGCs were slightly negative, were all smaller than .01 and none of the IGCs were significantly different than 0. Furthermore, a comparison of results corrected vs uncorrected for IGC yielded no difference. Thus, group randomization does not contribute to inflated IGC because the participants in each cohort are selected at random and do not share any common characteristic. Furthermore, sharing the same treatment group experience does not appear to have any effect on outcome either.

Based on this data, we do not plan on adjusting the sample size because of group randomization. We will check the IGC during preliminary analysis, and add into the model if it greater than 0.

11.4.6 Multiple comparisons

Using the current sample size of 90, we will still be able to detect a small sample size (d=.43 while correcting for up to 10 multiple comparisons with a Bonferroni correction (p=.05/10 =.005).

11.4.7 Minimum Sample Size

It is possible that recruitment will be slower and attrition higher than anticipated. Below are the detectable effect sizes with alternative sample sizes (at .80 power, p<.05, no adjustments)

- N=75 (25 per group) d=.37 (small)
- N=45 (15 per group) d=.48 (small, approaching medium)

11.5 Planned Interim Analyses (if applicable)

The Data and Safety Monitoring Board (DSMB) will meet quarterly to monitor safety. Aside from the usual safety monitoring, no interim analysis will be performed.
The treatments under investigation have been used widely (within MBCT) and piloted several times prior to the trial. Group differences in attrition, adherence and outcomes are part of the study questions, and do not pose threats to safety. See DSMB Section.

11.6 Missing Data

Based on the intent-to-treat principle, all subjects allocated to treatment will be included in primary analyses, with missing data imputed as described below.

Preliminary analyses will examine patterns of missing data and drop out rates (6, 7).To minimize missing data, I will attempt to gather follow-up information even if a subject drops out of treatment. However, if these analyses show major differences between groups in attrition or missing data, various methods for imputing data (e.g., maximum likelihood estimation, "worst case" analysis) will be explored to assess the degree to which different assumptions about missingness might affect the interpretation of results (8, 9). If results are similar regardless of method used, this will increase confidence in the results obtained. In addition to these efforts to preserve missing data, sample size calculations were made with an anticipated 15% attrition rate (2, 3, 10).

12. DATA HANDLING AND RECORD KEEPING

12.1 Types of Data:

Research material will be gathered through the following sources: interviews, questionnaires; audio recordings, laboratory based polysomonographic recordings and behavioral observations. All data will be collected for the purpose of this study and only used specifically for the research described in this application. There are no invasive procedures.

12.2 Data Confidentiality:

All study information will be kept in locked cabinets at the DPHB, with only study personnel having access to this information. All electronic recordings, laboratory measures, and written materials will be coded only with study identification numbers and the code that matches this number to participant names will be kept separately in a secure location. All data collected will be entered into an electronic database. Patient identifying information will be stored in a separate, password protected database.

12.3 Data Storage:

All data will be maintained on password-protected computers computers at the Department of Psychiatry and Human Behavior (DPHB), and backup copies will be made on a regular basis. All (paper) questionnaire data will be kept in a locked file cabinet in a different room from identifying and confidential information, which will be kept in a separate locked file cabinet. Identifying and confidential information about participants will not be entered or stored on the portable computers used to measure sleep and behavior and only study staff will have access to that data.

12.4 Data Verification and Review

The study staff will review all data collection forms on an ongoing basis for data completeness and accuracy as well as protocol compliance. Data will visually verified by pairs of research assistants who were not involved in data collection. The results of the ongoing data review will be incorporated into the Annual Report for the Independent Monitor(s).

12.5 Study Records Retention

Records will be retained according to regulations, as described in the IRB.
